# Comparison of naturalization mouse model setups uncover distinct effects on intestinal mucosa depending on microbial experience

**DOI:** 10.1093/discim/kyaf002

**Published:** 2025-02-01

**Authors:** Henriette Arnesen, Signe Birkeland, Harriet Stendahl, Klaus Neuhaus, David Masopust, Preben Boysen, Harald Carlsen

**Affiliations:** Department of Preclinical Science and Pathology, Faculty of Veterinary Medicine, Norwegian University of Life Sciences (NMBU), Ås, Norway; Department of Research, Østfold Hospital Trust, Sarpsborg, Norway; Faculty of Chemistry, Biotechnology and Food Science, Norwegian University of Life Sciences (NMBU), Ås, Norway; Department of Preclinical Science and Pathology, Faculty of Veterinary Medicine, Norwegian University of Life Sciences (NMBU), Ås, Norway; Core Facility Microbiome, ZIEL - Institute for Food & Health, Technische Universität München (TUM), Freising, Germany; Center for Immunology, Department of Microbiology and Immunology, University of Minnesota (UMN), Minneapolis, MN, USA; Department of Preclinical Science and Pathology, Faculty of Veterinary Medicine, Norwegian University of Life Sciences (NMBU), Ås, Norway; Faculty of Chemistry, Biotechnology and Food Science, Norwegian University of Life Sciences (NMBU), Ås, Norway

**Keywords:** naturalized mice, feralized mice, intestinal mucosa, memory T cells, gut microbiota

## Abstract

**Introduction:**

Concerns regarding the translational value of preclinical mouse models have been addressed by introducing various approaches of ‘naturalizing’ research mice, which provide them with more diverse microbiomes and physiological immune responses. We have previously shown that ‘feralized’ mice, that is, inbred laboratory mice raised in a farmyard-like, microbe-rich environment exhibit a shifted gut microbiota, matured immunophenotype, and reduced severity of colorectal cancer. Similar studies occasionally involve co-housing with wild or pet-store-raised mice as microbial donors integrating species-specific commensals and pathogens. To what extent these different practices of microbial exposure are crucial for the resulting mouse phenotype remains unclear.

**Methods:**

Here, we present the first side-by-side comparison of different methods to naturalize laboratory mice: co-housing with wild-caught house mice, feralization in a farmyard-like habitat only, or a combination of the two, with conventional clean laboratory mice as a reference.

**Results:**

Independent of the method, the naturalized colon-mucosa microbiota, was colonized by several *Helicobacter* species, and the colonic intestinal epithelial cells of naturalized mice displayed elevated expression of genes encoding antimicrobial peptides, mucus components, and reactive-oxygen-species-producing enzymes. They further showed significantly increased resident memory T cells in the colonic lamina propria and effector memory T cells in the mesenteric lymph nodes. The most pronounced changes of these parameters occurred in mice co-housed with wild-caught mice, while feralized mice displayed phenotypes that were intermediate between laboratory and co-housed mice.

**Conclusion:**

These findings enhance our understanding of naturalization model setups and effects on the gut barrier and immune system, thereby aiding future decisions on the utilization of naturalized mouse models.

## Graphical Abstract

**Graphical Abstract:**
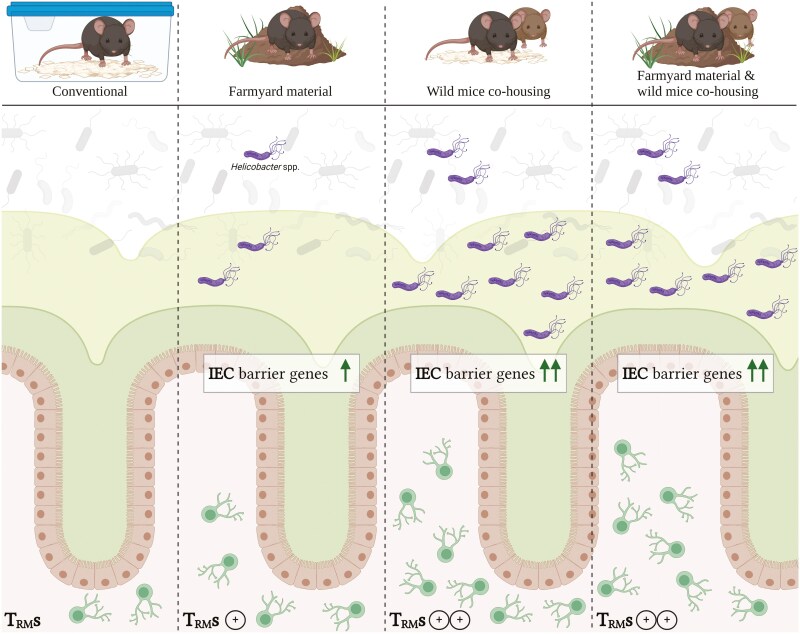

## Introduction

The house mouse (*Mus musculus*) inhabits a variety of microbially diverse environments, including both urban and rural areas close to humans and other mammals. Throughout evolution, the high microbial load of the environment has driven the adaptation of mammals, including the house mouse [1]. However, when mice are used as model organisms for humans, they are usually studied in perfect isolation from the outer, microbially diverse world. The hygienic housing of standard research mice, for example, specific pathogen free (SPF), creates an artificial microbial landscape and disrupt host–microbe interactions, potentially introducing bias in preclinical studies employing mice [2, 3].

Environmental stimuli have driven evolutionary imprints on mouse physiology, importantly through interactions with microorganisms. Both exposure to pathogens and beneficial microbes of the intestinal microbiota play a fundamental role in interacting with host cells to ensure the proper induction, training, and function of both the innate and adaptive immune system [4, 5]. The intestinal epithelial cells (IECs) influence the mucosal tissue inflammatory status by modifying the expression of genes involved in barrier function, antimicrobial defense, inflammation, immunosurveillance, and the production of reactive oxygen/nitrogen species (ROS/RNS) [6–11]. Furthermore, immune cells, such as resident memory T cells (Trms), accumulate and react in mucosal tissues upon microbial encounters [12]. Consequently, the development, differentiation, and function of various immune-cell populations residing in intestinal mucosa are modulated by the intricate crosstalk between gut microbiota, IECs, and immune cells. Stimuli are typically mediated by microbial antigens, metabolites, and pattern recognition receptor ligands, which may be sparse under hygienic laboratory conditions.

In recent years, various approaches have sought to ‘naturalize’ laboratory mice by bringing them closer to a natural setting with diverse microbial exposures, while keeping their traceable genetics [3]. Previous studies show substantial differences between laboratory and wild mice with respect to immune phenotypes and microbiological status [13–16], leading to naturalization attempts to elicit more realistic immune responses in mice to improve the translational potential of preclinical models [17]. Many diseases that are not adequately replicated in conventionally clean laboratory mice are now being studied using mouse models that include natural microbiota and/or pathogens [18]. We previously established a model system for housing laboratory mice in a naturalistic farmyard-type environment (called ‘feralization’), with or without the presence of wild house mice as host-specific microbial donors (co-housing). Mice feralized in a farmyard-like habitat displayed reduced colorectal tumor burden [19] as well as altered properties of the intestinal barrier [20] compared to hygienically reared equivalents, supporting a pivotal role of a natural context in improving accuracy in disease modeling in mice. In previous naturalization studies, both with and without co-housing with wild mice, increased immune cell maturation markers and altered microbiota profiles in the naturalized mice have been noted [17, 19, 21–23]. However, no direct comparisons of gut microbiota and immune parameters across methods have been conducted to date.

In this study, we therefore systematically compared the effects of three naturalization methods on gut microbiota, mucosal immune cell profiles, and gene expression in the IECs. We examined the feralization of laboratory mice in a farmyard-like habitat, co-housing with wild-caught house mice, and a combination of both. This study revealed distinct effects on intestinal mucosa depending on naturalization setup, with mice feralized in a farmyard-type habitat showing responses that were intermediate between clean laboratory mice and mice co-housed with wild mice. These findings suggest that the type of microbial exposure plays a significant role in shaping the resulting mouse phenotype.

## Methods and materials

### Animals and environmental settings

The feralization mouse model system was designed as previously described [19]. The indoor mouse pens contained sterile aspen woodchip bedding, mouse igloos, running wheels, and plastic boxes and tunnels allowing for sheltering and nesting.

**Figure 1.**
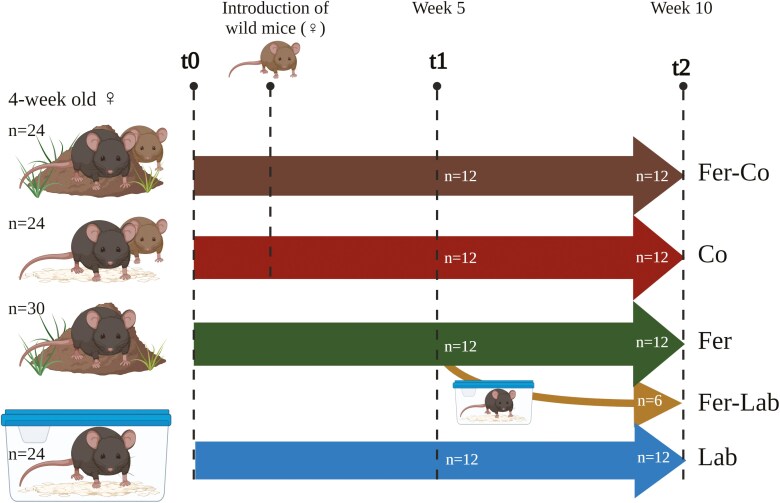
Experimental setup. Female mice born and weaned under conventional, clean laboratory conditions were randomized to groups housed under different conditions for 10 weeks: Wild-caught female mice (Wild, *n* = 9); B6 mice feralized in farmyard-type conditions, in the presence of wild-caught mice (Fer-Co, *n* = 24); B6 mice under clean conditions, in the presence of wild-caught mice (Co, *n* = 24); B6 mice feralized in a farmyard-type environment without any wild-caught mice present (Fer, *n* = 30); B6 mice from the Fer group returning to conventional, clean laboratory conditions half-way in the experiment (at 5 weeks, Fer-Lab, *n* = 6); and B6 mice in conventional, clean laboratory conditions (Lab, *n* = 24). Animals were euthanized and tissues were collected for analyses after 5 (t1) or 10 (t2) weeks. Number of animals euthanized at each timepoint are indicated.

Sixty-four three-week-old female C57BL/6JRj mice (B6; Janvier Labs, Saint-Berthevin Cedex, France) were acclimatized for 1 week under conventional, pathogen-free conditions in individually ventilated cages (IVCs; Innovive Inc., San Diego, CA) before being randomized to different experimental groups (Fig. 1), either housed in mouse pens or in IVCs. Two groups were feralized in mouse pens enriched with farmyard material. The environment was constructed to resemble a common habitat for the house mouse (*Mus musculus musculus*), consisting of organic soil, straw, and fecal content from organically farmed pigs, cows, and poultry. One of these feralized mouse groups was additionally co-housed with three wild mice (Fer-Co, *n* = 24). The other group was feralized in the absence of wild mice (Fer, *n* = 30), from which six mice were later moved to conventionally clean IVCs after 5 weeks of feralization (Fer-Lab, *n* = 6). A last group of mice was co-housed with three wild-caught mice in a mouse pen, but no farmyard material was provided (Co, *n* = 24). A control group was housed in conventionally clean IVCs (Lab, *n* = 24, 3 mice per cage). Sample size was determined by power analysis using JMP Power and Sample Size Calculator (https://www.jmp.com/) based on data from Ref. [20], where Power was set to 90%.

Wild mice (Wild) were caught at a farm in south-eastern Norway during September/October 2021 by overnight deployment of Ugglan Special No1 live traps (Grahnab, Gnosjö, Sweden), equipped with wood shavings, fresh grapes, biscuits, regular chow pellets, liver paté, and peanut butter as bait. Only female wild house mice (*M. m. musculus*) were introduced into the mouse pens and they were transferred immediately after capture. Wild mice were trapped for no more than 24 hours before release into pens. During the co-housing period, a total of four and five different female wild mice were introduced into the Fer-Co and Co pens, respectively. At any given time, three wild mice were present in each co-housing pen. Male wild mice (*n* = 9) were housed separately in cages, from which the bedding and feces were regularly distributed into the co-housed pens, thus adding donor material without risking fighting or unwanted mating. All the wild mice were represented for parasitology assessment as described below. All mice were individually marked by microchips injected subcutaneously (UNO PICO-ID ISO Transponder, 7 mm).

A separate group of animals from the same feralization setup were screened for murine viruses, as well as subjected to a parenteral influenza vaccine response assessment, performed after termination of the present experiment, reported separately [24]. All data presented in this manuscript are from original animal material and previously unpublished.

### Harvesting of tissues

Animals were randomized to two timepoints of harvesting—after 5 and 10 weeks of feralization (t1 and t2, respectively; Fig. 1). Tissues from 12 animals were harvested per group per timepoint, except from the six Fer-Lab mice that were collected at t2. At t1, a total of 48 animals were randomized to 4 days of harvesting, of which only Fer-Lab results are presented herein. At t2, a total of 60 animals were randomized to 5 days of harvesting. The animals were weighed and feces were collected. Subsequently, they were terminally anaesthetized by a single intraperitoneal injection (0.1 ml/10 g body weight) of ZRF-cocktail (Zoletil Forte (Virbac, Carros, France), Rompun (Bayer, Oslo, Norway), and Fentadon (Eurovet Animal Health, Bladel, The Netherlands) with the active ingredients zolezepam (32 mg/kg), tiletamin (32 mg/kg), xylazine (4.5 mg/kg), and fentanyl 26 µg/kg). Blood was collected by cardiac puncture while the animals were under terminal anesthesia. Tissues were collected after cervical dislocation.

Mesenteric lymph nodes (mLNs) and whole spleens were dissected and kept in RPMI-1640 medium (Sigma-Aldrich, #R0883) with 2% fetal bovine serum (FBS; Sigma-Aldrich, #F7524) on ice until extraction of cells for immunophenotyping. Mouse intestines were dissected and immediately put in ice-cold, sterile phosphate-buffered saline (PBS; Biowest, #L0615). First, 1 cm of ileums and mid 1 cm of colons, free of fecal pellets, were cut and collected for microbial community analysis. Ceca were removed, and the remaining small and large intestines were flushed with ice-cold PBS and Peyer’s patches removed. One-centimeter sections of distal jejunum and mid-to-distal colon were cut and collected for other purposes (not included here). The remaining tracts were cut longitudinally and kept in RPMI-1640 with PSEPx (100 units/ml penicillin + 100 µg/ml streptomycin (Biowest, #L0022), 25 µg/ml enrofloxacin (Sigma-Aldrich, #17849), and 100 units/ml polymyxin B (Sigma-Aldrich, #P4932)) on ice until isolation of lamina propria leukocytes and IECs.

### Parasitology

Fresh fecal pellets were collected from all experimental groups and examined for parasites by standard methods, as follows. For examination of endoparasites, a sucrose floatation test was conducted, while an immunofluorescence antibody test (IFAT) was conducted to assess *Giardia* spp. cysts and *Cryptosporidium* oocysts. The analyses were either conducted on individual samples or pooled samples from two to three individual mice.

### Microbial community analysis by 16S rRNA gene amplicon sequencing

Fecal pellets and intestinal tissues were collected in sterile tubes containing S.T.A.R. buffer (Roche Diagnostics, #03335208001). Samples were snap-frozen in liquid nitrogen immediately after collection and stored at −80°C until sample preparation. Sample preparation and high-throughput amplicon sequencing of the V3-V4 regions of 16S rRNA genes was conducted at the ZIEL Institute for Food & Health, Technical University of Munich, according to previously described procedures [25]. Briefly, a two-step PCR using the primer 341F (5′-CCT ACG GGN GGC WGC AG-3′) and 785R (5′-GAC TAC HVG GGT ATC TAA TCC-3′) was conducted for amplicon generation and sample barcoding. Sequencing was conducted on Illumina MiSeq machines. Fecal pellets were collected at baseline (before any microbial exposure, t0) and at termination (t1 or t2).

Raw reads were processed with the Integrated Microbial Next Generation Sequencing pipeline [26], producing denoised zero-radius operational-taxonomic units (zOTUs). Briefly, sequences were demultiplexed, trimmed to the first base with a quality score >3, and assembled. Sequences with <200 and >600 nucleotides as well as assembled sequences with expected error >3 were excluded from the analysis (USEARCH11 [27]), and zOTUs were calculated using the unoise3 pipeline of USEARCH. Sequences with abundance value <4 were discarded, and only zOTUs with a relative abundance >0.25% in at least one sample were kept. Non-16S rRNA gene sequences were removed by use of SortMeRNA (v4.2) [28] using SILVA release 128 as reference. Sequence alignment and taxonomic classification at 80% confidence level were conducted with SINA v1.6.1 [29] using the taxonomy of SILVA release 128. Phylogenetic tree was generated with RapidNJ [30]. The analyzed datasets included 3 403 239 quality- and chimera-checked sequences, representing a total of 1148 zOTUs. Sequencing depths were evaluated by rarefaction curves (observed zOTUs over the number of reads) to confirm suitability for subsequent analysis. Samples with inadequate sequence depths, even after their libraries were re-sequenced, were removed from further analyses (4 out of 59 colon mucosa samples, and 24 out of 60 SI mucosa samples). To compensate for differential sequencing depth between samples, read counts were normalized by simple division to their sample size followed by multiplication by the size of the smaller sample. Phylum names were manually refined to comply with the most recently validated phylum names [31].

Raw sequence files were deposited to the Sequence Read Archive under accession number PRJNA1116057.

### Isolation of cells for immunophenotyping and gene expression analysis

Cells from the mouse intestine were prepared and purified based on a previously published protocol [32]. Starting with removing mucus and separate the IECs, tissues were transferred into a Falcon tube with a dithiothreitol (DTT) solution (Hanks’ Balanced Salt Solution (HBSS, Sigma-Aldrich, #H9394), 10 mM N-(2-hydroxyethyl)piperazine-N′-(2-ethanesulfonic acid) (HEPES, Sigma-Aldrich, #H0887), 5 nM (ethylenedinitrilo)tetraacetic acid (EDTA, Sigma-Aldrich, #03690), 2% FBS, PSEPx, and 5 mM DTT (Sigma-Aldrich, #D9779)) and incubated with shaking at 175 rpm at 37°C for 15 min, before being passed through a 70-μm cell strainer. The tissue was kept for isolation of lamina propria cells as described below. The filtrate was collected for further isolation of IECs by two cycles of incubation with EDTA solution (same as DTT solution but without DTT) and straining as before. The filtrate was then centrifuged at 300*g* for 5 min at 8°C and the resulting cell pellet was washed twice by resuspending in cold HBSS with 2% FBS and centrifuged as before. The suspension was divided into two separate tubes before the last centrifugation, followed by resuspending the cell pellet in either 5 ml cRPMI (RPMI-1640 w/ 25% HEPES + 10% FBS + 2 mM Alanyl-glutamine (Sigma-Aldrich, #G8541) + 1 mM sodium pyruvate solution (Sigma-Aldrich, #S8636) + 1× Minimal Essential Medium Non-essential Amino Acid Solution (Sigma-Aldrich, #M7145) + PSEPx) and stored on ice for subsequent immunophenotyping, or RNA*later*™ Stabilization Solution (Invitrogen, #AM7021; 1 ml for colon and 2 ml for small intestine cell pellets), incubated overnight at 4°C and then stored at −80°C until RNA isolation.

The remaining tissue was added to a Falcon tube containing pre-digest solution (HBSS with Ca^2+^ and Mg^2+^, Sigma-Aldrich, #H9269; PSEPx; 10 mM HEPES) for removal of enzyme inhibitors and incubated with shaking at 200 rpm at 37°C for 10 min. Tissue was retrieved by pouring onto a sterile, crude metal sieve, and moved to a Petri dish with enzyme solution (pre-digest solution with 200 Kunitz units/ml DNase I (Sigma-Aldrich, #D5025), 0.5 mg/ml collagenase D (Roche, #11088866001) and 3 mg/ml dispase II (Sigma-Aldrich, #D4693)). The tissue was minced with sterile scissors and incubated at 37°C for 10 min before 0.5x volumes cRPMI was added to stop tissue digestion. The tissue was then triturated with an 18G needle, filtered through a 70-μm cell strainer, and centrifuged at 300*g*, 8°C for 5 min. The supernatant was discarded, and the collected cells were washed twice with HBSS with 2% FBS. After the last wash, the cell pellet was resuspended in cRPMI and stored on ice until immunophenotyping.

From mLNs and whole spleens, cells were extracted using GentleMACS dissociator (Miltenyi). For splenic tissue, a collagenase/DNAse solution (1 mg/ml and 600 KU/ml, respectively) was used for digestion and suspensions were additionally treated with 3 ml ACK Lysing Buffer (Gibco™, #A1049201) for 1 min to lyse erythrocytes. Single-cell suspensions were prepared by running through a 70-µm cell strainer (BD Biosciences), and cells were washed twice using RPMI-1640 + 2% FBS. Concentrations of single-cell suspensions were standardized by trypan blue cell counts using Countess II automated cell counter (Thermo Fischer Scientific).

### Immunophenotyping

Immunophenotyping was carried out on ice by incubating single-cell suspensions in RPMI-1640 with 0.5% bovine serum albumin. Following Fc blocking with anti-CD16/CD32 antibody, cells were stained with Zombie NIR™ Fixable Viability Dye or LIVE/DEAD™ Fixable Aqua Dead Cell Stain (Biolegend #423105, or Thermo Fisher #L34957, respectively) and incubated with combinations of monoclonal antibodies listed in Supplementary Table S1. For intracytoplasmic staining, surface staining was followed by additional steps of treatment with Intracellular Fixation & Permeabilization buffer (eBioscience™ #88-8824-00) or Foxp3 Staining buffer (eBioscience™ #00-5523-00) according to manufacturer’s manual. Cells were analyzed using a Cytoflex LX flow cytometer and Kaluza 2.2 software (Beckman Coulter). Gating strategies are depicted in Supplementary Fig. S1.

### *Ex vivo* activation of immune cells

Cells isolated from mLNs were seeded on 96-well plates (500 000 cells/well) in triplicates. The cells were incubated with a cocktail of brefeldin A (10 μg/ml; Sigma-Aldrich #B7651) in RPMI-1640 medium with PMA (phorbol 12-myristate-13-acetate, 50 ng/ml; Sigma-Aldrich #P8139) and ionomycin (750 ng/ml; Invitrogen™ #I24222) for T cell activation. Cells were incubated for 4 h, spun down, and stained for immunophenotyping of intracytoplasmic IFNγ as described above.

### RNA isolation

Total RNA was extracted from the isolated IECs using the NucleoSpin RNA Mini kit (Macherey-Nagel) following the manufacturer’s manual. RNA concentration and purity was measured using NanoDrop™ 2000 (Thermo Scientific™). Impure samples (260/280 and/or 260/230 ratios < 1.8) were rinsed for removal of possible contaminants using a procedure based on previously described protocols [33, 34]. Briefly, RNA was re-precipitated with 0.1x volumes 3 M sodium acetate and 2.5x volumes 70% ethanol, mixed well, and incubated on ice for 30 min. RNA was then pelleted by centrifugation at 21 000*g* for 15 min at 4°C, followed by pipetting off supernatant, washing with ethanol, and centrifuging as before. After supernatant removal, residual ethanol was left to evaporate in a fume hood at room temperature. Pellet was loosened by gently tapping the tube before RNA was resuspended in RNase-free H_2_O. All re-precipitated samples were of suitable quality according to new measurements with NanoDrop™ 2000 and diluted before storage at −80°C. RNA integrity of the samples was assessed using the RNA 6000 Nano kit with Agilent 2100 Bioanalyzer (Agilent Technologies) prior to gene expression analysis (Supplementary Fig. S2).

### Primer assay design and validation

Selection of target genes for a gene expression assay representing the intestinal barrier was based on previous findings from RNA-sequencing [20] and further searches in the literature to extend the number and functions of relevant targets. Gene targets were chosen by fulfilling the following criteria: (1) expression proven in murine IECs; (2) expression level affected by microbial exposure or intestinal inflammation; and (3) gene function is related to at least one of the following categories: physical barrier, antimicrobial peptides, immunosurveillance, inflammation, and ROS/RNS production. Specific primers spanning exon-exon-junctions were designed with D3 Assay Design (Standard BioTools Inc.; https://d3.standardbio.com/). Primer sequences are listed in Supplementary Table S2.

For primer assay validation, total RNA was reverse transcribed into complementary DNA (cDNA) with iScript cDNA synthesis kit (Bio-Rad Laboratories Inc.) following the manufacturer’s protocol. Quantitative PCR (qPCR) was then performed with different dilutions of pooled small intestine and colon IEC cDNA using Ssofast Evagreen Supermix (Bio-Rad Laboratories Inc.) on Bio-Rad CFX96 Touch Real-Time PCR Detection System (Bio-Rad Laboratories Inc.) (enzyme activation at 95°C (1 min) and 40 cycles of denaturation at 96°C (5 s) and annealing and elongation at 60°C (20 s)). Primer specificity was assessed by melting curve analysis in CFX Maestro Software (Bio-Rad Laboratories Inc). Efficiencies (E) were estimated in LinRegPCR analysis software [35]. Primer pairs showing one specific melt peak and Ê and *R*^2^ within the acceptable range (Ê = 2 ± 0.1 and *R*^2^ > 0.98) were included in further analyses.

### Gene expression analysis

Relative expression levels of genes in the intestinal barrier assay were measured by high-throughput microfluidic RT-qPCR with the Biomark™-system (Standard BioTools Inc.) following the manufacturer’s protocol. All reagents and instruments were provided by Standard BioTools Inc. unless stated otherwise. Briefly, cDNA was synthesized from total RNA (250 ng) using Reverse Transcription Master Mix, and pre-amplified in a thermal cycler using a pool of the 41 validated primer pairs and Preamp Master Mix (12 cycles). Samples were cleaned by Exonuclease I treatment (4 U/μl, Thermo Scientific) and diluted 5-fold (small intestine samples) or 10-fold (colon samples). 96.96 (colon samples) and 48.48 (small intestine samples) Dynamic Array Integrated Fluidic Circuits (IFCs) were primed with Control Line Fluid in the IFC Controller HX (for 96.96 IFC) or MX (for 48.48 IFC). Primer pairs were combined with TE-buffer (Macherey-Nagel) and Assay Loading Reagent (2×), while diluted samples were mixed with DNA Binding Dye Sample Loading Reagent (20×) and SsoFast EvaGreen Supermix (Bio-Rad Laboratories Inc.) and added to the wells on the IFC and loaded in the respective Controller. Finally, the IFCs were inserted into Biomark™ HD for qPCR measurements (program as described for the primer validation).

### Statistical analysis

**Figure 2.**
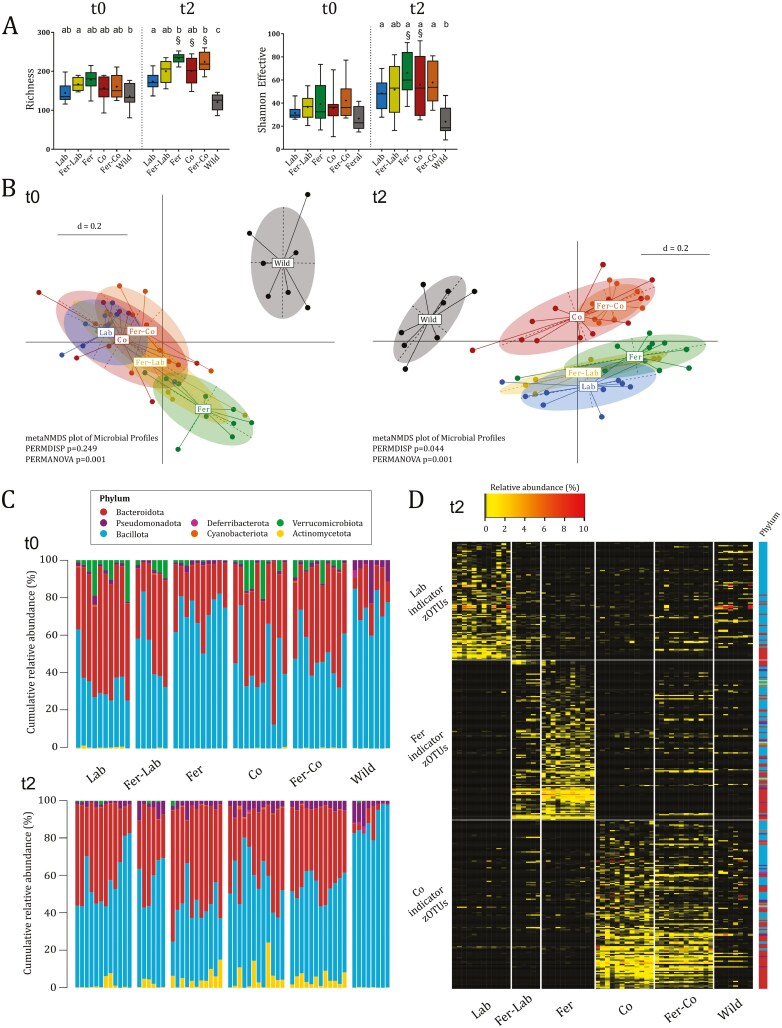
The fecal microbiota composition changed significantly both following feralization in a farmyard-like habitat and co-housing with wild mice. Fecal microbiotas were characterized at baseline (t0) and following 10 weeks of feralization in the presence or absence of wild mice (t2). (A) Richness (observed number of zOTUs) and Shannon effective counts. Box plots show median (line), mean (+), IQR (box), and minimum to maximum (whiskers). Letters designate significant (*P* ≤ 0.05) differences between groups at each timepoint determined by two-way ANOVA and Tukey’s multiple comparisons tests following significant ANOVAs. Section sign designates significant (*P* ≤ 0.05) over-time difference within groups. Absence of letters indicate no significant differences detected. (B) Non-metric multi-dimensional scaling (NMDS) plot of fecal microbiota profiles (generalized UniFrac distances) at baseline (t0) and endpoint (t2). Significance of separation was determined by PERMANOVA, and dispersion of groups determined by PERMDISP. d = distance scale. (C) Taxonomic binning at phylum level, presented as relative abundance across groups at baseline (t0) and endpoint (t2). (D) Heatmap of abundance of Lab-, Fer-, and Co-associated zOTUs identified by indicator species analysis. Relative abundances <0.25% are represented by black color. Phyla of which the zOTUs belong are designated with colored squared as specified in C. See also Supplementary Table S3A. All plots: Lab *n* = 10; Fer-Lab, *n* = 6; Fer, *n* = 10; Co, *n* = 10; Fer-Co, *n* = 10; Wild, *n* = 7.

**Figure 3.**
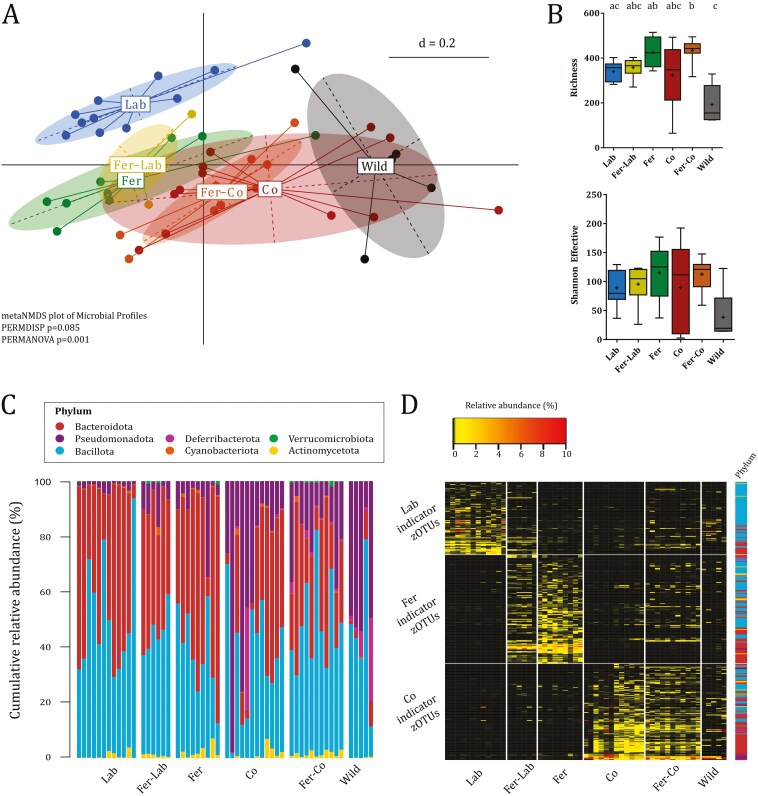
Mucosal-associated microbiota composition in mice colons were significantly different dependent on type of naturalization at endpoint (t2). (A) NMDS plot of mucosa-associated microbiota profiles (generalized UniFrac distances). Significance of separation was determined by PERMANOVA, and dispersion of groups was determined by PERMDISP. d = distance scale. (B) Richness (observed number of zOTUs) and Shannon effective counts. Box plots show median (line), mean (+), IQR (box), and minimum to maximum (whiskers). Letters designate significant (*P* ≤ 0.05) differences between groups at each timepoint determined by two-way ANOVA and Tukey’s multiple comparisons tests following significant ANOVAs. Absence of letters indicate no significant differences detected. (C) Taxonomic binning at phylum level, presented as relative abundance across groups at endpoint (t2). (D) Heatmap of abundance of Lab-, Fer-, and Co-associated zOTUs identified by indicator species analysis. Relative abundances <0.25% are represented by black color. Phyla of which the zOTUs belong to are designated with colored squared as specified in C. See also Supplementary Table S3B. All plots: Lab, *n* = 12; Fer-Lab, *n* = 6; Fer, *n* = 9; Co, *n* = 12; Fer-Co, *n* = 11; Wild, *n* = 5 (3 females and 2 males).

Microbial profiles and composition were analyzed in the R programming environment (R v4.2.2) [36] using Rhea (available from https://lagkouvardos.github.io/Rhea/) [37]. To identify patterns of differentially abundant and prevalent zOTUs across the feralization types we conducted an indicator species analysis implemented by the *indicspecies* package [38] in R. The indicator species algorithm was developed by Dufrene and Legendre and has been employed by us previously to track the persistence of OTUs in mice following feralization [21, 39]. The significance of the associations was determined by permutation tests followed by Benjamini–Hochberg correction of resulting *P* values. The closest species related to the indicator-zOTUs sequences were identified with EzBioCloud [40]. See Supplementary Table S3 for a complete list of the indicator-zOTUs presented in Figs 2D and 3D. To explore whether taxa identified as indicator species were unique for the different feralization setups, we gave all indicator zOTUs species names based on the closest sequence similarities identified with EzBioCloud and plotted Venn diagrams of the species names using BioVenn [41].

Fold-change (FC) values were obtained from the gene expression data using the Livak method [42]. Quantification cycle (Cq) values for each target gene within a sample were normalized by subtraction of the average of the given samples’ housekeeping genes (HKG) *Tbp* and *Gapdh*, yielding ΔCq values. For each ΔCq, a corresponding ΔΔCq value was calculated by subtracting the average ΔCq of the control group (Lab) for the given target. To avoid bias introduced by the exclusion of runs that lacked signal (very low RNA levels/failed run), ΔCq values were imputed for the relevant samples by subtracting the HKG average from 30 (the maximum number of cycles). Finally, FC was calculated as two raised to the power of -ΔΔCq (2^-ΔΔCq^). Statistical tests comparing the experimental groups were performed for each gene target individually using log_2_FC values in GraphPad Prism 10 v10.1.2. Gene expression data was tested by ordinary one-way ANOVA if residuals were normally distributed (Shapiro–Wilk test) and data had equal variance (Brown–Forsythe test), Kruskal–Wallis in case of non-normal distributed residuals, or Brown–Forsythe ANOVA in case of unequal variance. *P* values were corrected for multiple comparisons using Tukey’s, Dunns, or Dunnett’s T3 test, respectively. Significance threshold was set to *α* < 0.05. Heatmaps were generated with the *heatmap.2* function from the *gplots* package (version 3.1.3.1) in R. See Supplementary Table S5 for all results from statistical analyses of gene expression data.

## Results

### Changes in fecal and mucosal microbiota profiles depended on naturalization setup

A gross parasitological examination of fecal samples revealed the presence of parasitic eggs and cysts in mice co-housed with wild mice, the wild mice themselves, and a single occurrence of *Giardia* cysts in Fer mice (Supplementary Table S4). We had previously detected no parasites in feralized mice in the absence of wild donor mice [19]. No further speciation of the cysts was performed, and whether the cysts originated from donor mice or domestic animal excrements could thus not be determined. A serological assessment for viruses in separate mice originating from the present setup was published previously [24], indicating exposure of several common murine viruses in wild mice, transfer of most of these in co-housed mice but no detection in feralized mice.

The fecal microbiota composition before naturalization (baseline, t0) was comparable across the experimental groups, while the wild mouse fecal microbiota showed a distinct profile characterized by lower relative abundance of Bacteroidota and high relative abundances of Bacillota and Pseudomonadota (Fig. 2A–C). Members of Verrucomicrobiota were only detected in a few wild mice (3/6) and the relative abundances were below cutoff for analyses (<0.25%). However, they were detected in all individuals of the experimental groups, with one exception (in the Co group), with relative abundances ranging from 0% to 22%, attributed to *Akkermansia* spp. The higher relative abundance of Pseudomonadota in the wild mice was largely attributed to *Helicobacter* spp. composing 1.5–4.3%. The higher relative abundance of Bacillota in wild mice was mainly attributed to *Lactobacillus* spp. and members of the *Clostridiales vadin* BB60 group.

After 10 weeks of microbial exposure (endpoint, t2), the shifts in fecal microbiota profiles depended on the type of naturalization. The overall fecal microbiota profiles showed a clear clustering of the Lab, Fer-Lab, and Fer groups away from the wild mice, and a cluster of the Co and Fer-Co groups towards the wild mice (Fig. 2B). The richness of Fer, Fer-Co, and Co groups increased significantly over time, and at endpoint, the Fer and Fer-Co group had significantly higher richness compared to Lab, while due to high variance within the Co group, no significant difference was established (Fig. 2A). The wild mice had significantly fewer unique zOTUs compared to the experimental groups (Fig. 2A). No significant differences were detected in Shannon effective numbers between the experimental groups, while the wild mice had significantly lower numbers compared to the Lab, Fer, Co, and Fer-Co groups, indicative of a microbiota characterized by fewer dominating species. Feralized mice, both in the presence and absence of wild mice, showed a bloom of Actinomycetota and Pseudomonadota, attributed to *Bifidobacterium* spp. and *Helicobacter* spp. (Fig. 2C). At this timepoint, members of the Actinomycetota were only detected above threshold for analysis in half of the wild mice (4/8) at very low abundance (0.32–0.48%) and were attributed to *Coriobacteriaceae UCG-002* rather than *Bifidobacterium* spp.

To elaborate on potential microbes derived from the farmyard-like environment versus wild mice co-housing, we conducted an indicator species analysis on the endpoint (t2) data to identify zOTUs that were associated with the Lab, Fer, and Co mice. The indicator species analysis of fecal microbiota showed 105, 142, and 150 zOTUs that were only indicative of the Lab, Fer, and Co groups, respectively. By plotting the relative abundances of these indicator zOTUs for all groups, we could track the zOTUs associated with the laboratory baseline (Lab indicator zOTUs), the farmyard-like environment (Fer indicator zOTUs), and co-housing with wild mice (Co indicator zOTUs) in all groups. The Fer-Co group had a high abundance of Co-associated zOTUs but few Fer-associated zOTUs, suggesting a stronger effect of co-housing over the farmyard-like environment on the fecal microbiota (Fig. 2D).

By plotting lists of the closest sequence-similarity species names of the zOTUs indicative of the Lab, Fer, and Co groups in a Venn diagram, we had a closer look at unique and overlapping taxa within the indicator zOTUs (Supplementary Table S3C). In this analysis, we found 17, 36, and 30 unique taxa for Lab, Fer, and Co, and 5 (Lab and Fer), 5 (Lab and Co), and 10 (Fer and Co) taxa shared between two groups only. The taxa found unique to the farmyard-like environment included *Eubacterium* spp., *Phocaeicola vulgatus* (phylum Bacteroidota), and *Rhodospirillum rubrum* (phylum Pseudomonadota), while the taxa unique for co-housing with wild mice included *Odoribacter splanchnicus* (phylum Bacteroidota), *Clostridium leptum* (phylum Bacillota), *Turicimonas muris* (phylum Pseudomonadota), *Mucispirillum schaedleri* (phylum Deferribacterota), *Granulimonas faecalis* (phylum Actinomycetota), *Helicobacter typhlonius*, and *Helicobacter aurati* (phylum Pseudomonadota). The overlapping taxa between Fer and Co included *Helicobacter ganmani* (phylum Pseudomonadota), *Vampirovibrio chlorellavorus* (phylum Cyanobacteriota), and *Parasutterella excrementihominis* (phylum Pseudomonadota), suggesting that these species are derived from both a farmyard-like environment and co-housing with wild mice.

Collectively, our data highlight specific candidate species likely introduced from either the farmyard material or through co-housing with wild mice. In addition, these findings underscore the differences in microbiota composition between the two naturalization methods.

Similar to the endpoint fecal microbiota, the mucosal-associated microbiota in colon showed shifts according to type of naturalization, where the co-housed groups clustered in direction of the wild mice (Fig. 3A). Fer-Co had significantly higher richness than Lab, while Fer and Fer-Co groups had significantly higher richness than Wild. No significant differences were detected for Shannon effective numbers (Fig. 3B). The mucosal-associated microbiota further indicated taxa derived from the environment or contact with wild mice. Relative abundances of Bacteroidota were higher in Lab mice, as well as mice feralized in the absence of wild mice (Fer and Fer-Lab) (Fig. 3C). This was largely due to higher relative abundances of *Alloprevotella* spp, *Prevotellaceae UCG-001*, unknown *Muribaculaceae*, *Bacteroides* spp. (in Fer and Fer-Lab, not in Lab), and *Odoribacter* spp. (in Fer and Lab, not in Fer-Lab). Deferribacterota was detected (above cutoff for analysis) in most co-housed and all wild mice, while only one Fer-mouse carried this phylum. This suggests that Deferribacterota, represented by *Mucispirillum schaedleri*, is more readily transferred from wild mice than from the environment. Relative abundance of Pseudomonadota, predominantly attributed to *Helicobacter* spp., was greater in all naturalized mice compared to Lab, but to a higher level in the Co, Fer-Co, and wild mice. Moreover, the levels of *Helicobacter* spp. in the mucosa-associated microbiota were higher than in the fecal microbiota, on average 4.9–28.4% in Fer-Lab, Fer, Fer-Co, and Co, and 41.1% in Wild compared to 0.9–7.1% in feces in the respective groups. Thus, the considerable bloom of this phylum in the mucosa is likely conferred by both farmyard material and wild mice co-housing, with wild mice as the major source.

We also conducted an indicator species analysis on the mucosal-associated microbiota in colon, to identify zOTUs associated with the Lab, Fer, and Co mice. The indicator species analysis showed 93, 139, and 123 zOTUs that were only indicative of the Lab, Fer, and Co groups, respectively (Fig. 3D). The Fer-Co group showed a high abundance of Co-associated zOTUs and to a lesser extent Fer-associated zOTUs, supporting a stronger effect of co-housing versus feralization on the mucosal-associated colonic microbiota in this experiment.

Bacterial species with the closest sequence similarity to the indicator zOTUs for each of the groups Lab, Fer, and Co were plotted in a Venn diagram to identify unique and overlapping taxa among the indicator zOTUs (Supplementary Table S3D). We found 17, 27, and 26 unique taxa for Lab, Fer, and Co, respectively, and 8 (Lab and Fer), 2 (Lab and Co), and 10 (Fer and Co) taxa shared between two groups only. The unique taxa for the farmyard-like environment included *Eubacterium* spp.*, Ruminococcus* spp.*, Clostridium lentum*, *Butyricicoccus porcorum*, *Velocimicrobium porci*, *Eisenbergiella porci* (phylum Bacillota), *Phocaeicola vulgatus* (phylum Bacteroidota), and *Rhodospirillum rubrum* (phylum Pseudomonadota). As for feces, the overlapping taxa between Fer and Co included *Parasutterella excrementihominis* (phylum Pseudomonadota), *Vampirovibrio chlorellavorus* (phylum Cyanobacteriota), and *H. ganmani* (phylum Pseudomonadota), in addition to *Acetatifactor muris* (phylum Bacillota) and *Mucispirillum schaedleri* (phylum Deferribacterota), indicating these species may be derived from the farmyard-like environment as well as co-housing with wild mice. The taxa unique for co-housing with wild mice included *Odoribacter splanchnicus*, *Bacteroides* spp. (phylum Bacteroidota), *Granulimonas faecalis* (phylum Actinomycetota), *Turicimonas muris* (phylum Pseudomonadota), *Helicobacter cinaedi*, *Helicobacter typhlonius*, and *Helicobacter aurati* (phylum Pseudomonadota).

The gut microbiota of microbially experienced mice is considered stable over time, as reported in ‘wildling’ mice [22]. Thus, we addressed whether the gut microbiota of feralized mice remained stable despite relocation back to a hygienic conventional laboratory setting, referred to as ‘discontinued’ feralization (Fer-Lab). Although relocating Fer mice to clean conditions shifted microbiota composition towards Lab-mice, the Fer-Lab mice retained a distinct microbiota composition in between that of Fer mice and Lab mice (Supplementary Fig. S3A–C).

Mucosal-associated microbiota composition in the mice jejunums also significantly differed dependent on naturalization setup (Supplementary Fig. S4A–C). However, poor sequencing quality due to high host-bacterial DNA ratio rendered 24 out of 60 jejunum samples unsuitable for further analyses and, consequently, a lower sample number for the analysis of this tissue compared to the fecal samples and colon tissue.

### Co-housing with wild mice led to accumulation of memory T cells within the colon lamina propria and in mesenteric lymph nodes

To characterize the influence of the various naturalization setups on the mouse immunophenotypes, we measured relative proportions of cellular phenotypes in cells isolated from lamina propria and mLNs by use of flow cytometry gating strategies as shown in Supplementary Fig. S1.

**Figure 4.**
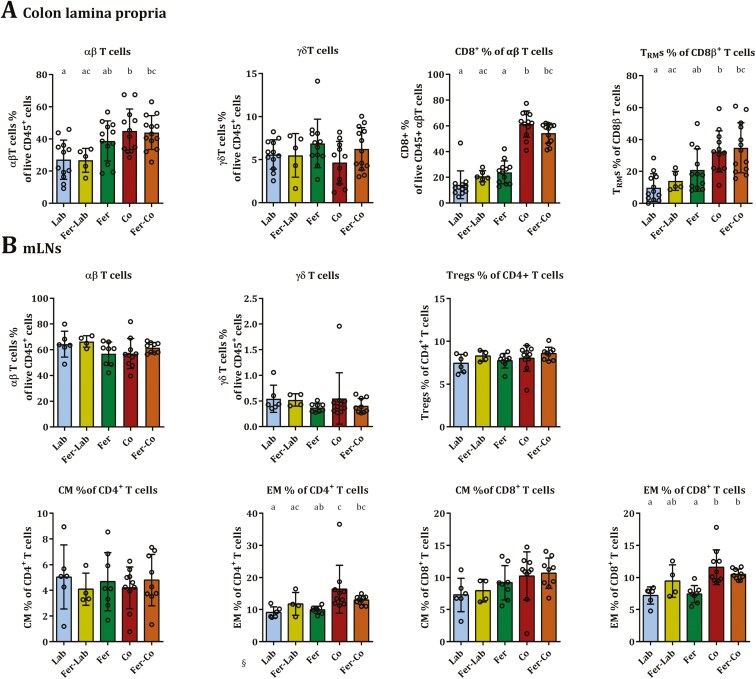
Phenotypic markers of T cell subsets in (A) colon lamina propria and (B) mesenteric lymph nodes. Tissue-resident memory T cells were defined as CD103^+^CD69^+^CD8β^+^αβTCR cells. Gating strategies are presented in Supplementary Fig. S1. All graphs are presented as mean, with the standard deviation (SD) shown by whiskers. Different letters indicate statistical significance at alpha level 0.05 as determined by one-way ANOVA followed by Tukey’s multiple comparisons tests in cases of significant ANOVA, or non-parametric Kruskal–Wallis test followed by Dunn’s multiple comparisons tests in case of significant Kruskal–Wallis. Non-parametric statistical methods were applied to the graphs marked §. Absence of letters indicate no significant differences detected. Lab, *n* = 6; Fer-Lab, *n* = 4; Fer, *n* = 8; Co, *n* = 10; Fer-Co, *n* = 9. See also Supplementary Fig. S5.

In the colon lamina propria, proportions of αβ T cells, CD8+ T cells, and tissue-resident memory T (Trm) cells, defined as CD103^+^CD69^+^CD8β^+^αβTCR^+^ cells [43], were significantly higher in co-housed mice (Co and Fer-Co), compared to the other experimental groups (Fig. 4A). In the small intestinal lamina propria, αβ T cells were similarly increased in the co-housed groups, but Trm cells were sparsely detected and not significantly different (Supplementary Fig. S5A). In mLNs, we detected significantly higher proportion of effector memory T cells in both CD4 and CD8 compartments in co-housed mice compared to the Lab mice, but not in Fer mice, while no significant differences were detected in either αβ T cells, γδ T cells, Tregs or central memory T cells (Fig. 4B).

We assessed subsets of B cells (defined as B220^+^ Live CD45^+^ cells) in the mLNs; B1 defined as CD5^+^CD11b^−^ B cells and B2 cells defined as CD5^-^CD11^−^ B cells. Moreover, the expression of CD38 as a marker of B cell activation [44] was measured on B1 and B2 cells. However, we found no significant differences in the relative numbers of these B cell subsets in mLNs across the groups (Supplementary Fig. S5B).

To address if different setups of naturalization affected immune function, we examined IFNγ production of T cells from mLNs in response to *ex vivo* stimuli. Our findings revealed no significant effects of microbial exposure on IFNγ production in T cells in this experiment (Supplementary Fig. S5C).

### Expression of mucosal barrier genes in intestinal epithelial cells was more altered by co-housing with wild mice than by feralization

**Figure 5.**
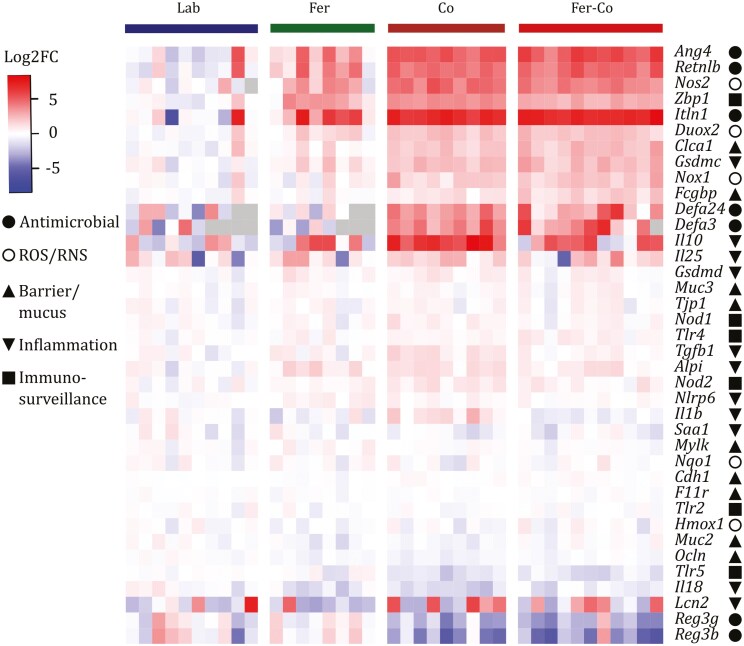
Heatmap of gene expression levels in colonic epithelial cells measured by high-throughput microfluidic RT-qPCR. Heatmap shows log2 fold change values for all measured genes, grouped according to experimental groups. Gray areas indicate missing datapoints. General function is indicated by symbols: filled circle, antimicrobial; circle, ROS/RNS; triangle, barrier/mucus; upside-down triangle, inflammation; and square, immunosurveillance. Significant differences were determined by either one-way ANOVA, Kruskal–Wallis test, or Brown-Forsythe test, followed by Tukey’s, Dunn’s, or Dunnett’s T3 multiple comparisons tests in cases of significant ANOVA, respectively (see Supplementary Table S5).

A targeted gene expression analysis of mRNA from IECs isolated from the mouse intestines revealed significantly altered relative expression of several target genes dependent on type of naturalization. In total, 27 out of 38 genes were differentially expressed in colons of naturalized mice compared to lab mice (Supplementary Table S5). The small intestine displayed 20 out of 37 differentially expressed genes, 15 of which were in common with the colon (Supplementary Table S5). Co-housing with wild mice initiated the greatest shift in IEC gene expression profiles in both intestinal segments, particularly in the colon (Fig. 5). The most affected genes encode proteins critical to the gut barrier integrity, including antimicrobials (including *Ang4*, *Retnlb*, *Reg3g*, and *Reg3b*) [45–47], ROS production (*Duox2* and *Nox1*) [8, 11], and proteins involved in mucus structure and rearrangement (*Clca1* and *Fcgbp*) [48, 49] (Fig. 5; Supplementary Table S5).

The relative expression of *Zbp1* associated with viral responses [50] was higher in the colons of all naturalized groups, and in the small intestine of co-housed mice, compared to Lab mice. Finally, *Gsdmc*, a gene related to helminth responses [51], was higher in the colons of all naturalized groups compared to Lab. The IEC expression-patterns in the small intestine were generally comparable to those in the colon (Supplementary Fig. S6).

In conclusion, changes in gene expression related to mucosal barrier integrity in IECs from both the small intestine and colon depended on the type of naturalization, with the greatest modulation observed in co-housed mice. Overall, the Fer mice expression profile appeared as an intermediate between Lab and co-housed mice.

## Discussion

We have established a model system for feralizing mice, in absence or presence of wild mice, which greatly influences mouse gut immunity and microbiota composition [19–21]. To further evaluate this model of naturalizing mice, the current study aimed to determine whether different feralization approaches differentially affect the immune system and microbiota composition of laboratory mice. Employing various setups of the model allowed us to assess the relative impact of environmental microbial exposure from a naturalistic habitat, host-specific microbial transfer from wild mice, or a combination of both.

Microbiota profiling of feces and mucosa revealed shifts in profiles that depended on the type of naturalization setup. By applying an indicator-species analysis to the microbiota data from our current study, we identified specific microbes that overlap between groups, and species that likely derived from a laboratory environment, farmyard-like environment, or co-housing with wild mice. Amongst the microbes likely derived from the farmyard-like environment, we detected several associated with positive effects in the gut, such as known butyrate producers [4, 52, 53], and species reported to prevent growth of pathogens [54, 55] and inflammatory conditions in the mouse gut [56, 57]. Overall, these findings indicate a potential positive modulatory effect of feralization on gut microbiota. In addition, in colon mucosa, we identified taxa isolated from pigs, including *B. porcorum*, *Velocimicrobium porci*, and *E. porci*, likely derived from porcine droppings within the farmyard material. However, literature on these novel species and possible host effects is limited.

Both in feces and mucosa, *H. ganmani* was associated with both the farmyard-like environment and co-housing with wild mice. Meanwhile, additional *Helicobacter* species were specifically associated with co-housing (*Helicobacter aurati*, *Helicobacter cinaedi*, and *Helicobacter typhlonius*), suggesting they were likely acquired from the wild mice. These results support our previous findings from fecal samples of wild mice captured in the same geographical area [21], and previous feralization experiments [19], further implying that *Helicobacter* ssp. readily engraft from microbially rich environments. Prevalence of *Helicobacter* in wild mice has previously been reported [58], where members of the genus were detected in 100% of wild mice captured. *H. ganmani* and *H. typhlonius* were most prevalent, aligning with our identification of these as unique species in co-housed mice. Moreover, enrichment of SPF-mouse microbiota with *Helicobacter* spp. confers protection against enteric infection [59] and anti-tumor immunity with long-term control of colorectal cancer [60]. The former study also identified *Helicobacter* spp. as frequent colonizers in wild mice, corroborating our wild mouse microbiota analyses. Interestingly, *Helicobacter* spp. appears to readily colonize the colonic mucus, which may indicate a significant role for these bacteria in host barrier interactions.

Analyses of immune competence showed higher proportions of αβ T cells, particularly attributed to CD8+ T cells, and of these, increased proportions of Trm phenotype (CD103^+^CD69^+^) in co-housed mice. A similar trend was observed in Fer mice, but not significantly different from Lab mice. Absolute counts were not collected in this study, but seen together, the increase of Trms in co-housed mice appeared relatively large. This is the first time Trms have been studied in these naturalization setups, and our findings are in support of related studies, such as a Trm deficit demonstrated in SPF mice that was restored after co-housing with pet shop mice [23]. Furthermore, our observation of a higher proportion of effector memory T cells in both CD4 and CD8 compartments in co-housed mice mLNs supports an enhanced immune competence following exposure of wild-mice microbes. The stronger effects observed following co-housing with wild mice compared to feralization alone suggest that a mouse-to-mouse transfer of microbes is more potent in enhancing immune responses of the recipient, perhaps due to the transfer of pathogens, corroborating results from previous studies of co-housing with pet shop mice [23, 61].

The exact consequences of elevated memory T cells and Trms are still not completely understood, but both cell types comprise important parts of the immune sensing network, monitoring local disturbances and swiftly responding to and conferring protection against infection [61] and cancer [12, 62]. For instance, increased proportions of memory T cells following co-housing with pet shop mice conveyed protection against *Listeria* infection, yet rendered no effect on *Salmonella* nor mucosal infection with *Chlamydia muridarum* [61].

To assess whether different naturalization setups differentially impacted the epithelial barrier, we assessed the relative expression of specific genes in IECs. By isolating the IECs specifically, we were able to more precisely identify changes in gene expression within the epithelial monolayer that forms the intestinal barrier. Our findings indicate that naturalization significantly influences gene expression in colonic IECs, with a more modest effect observed in the small intestine. Co-housing with wild mice led to more pronounced alterations in expression levels compared to mice feralized in a farmyard-like setting only.

The relative expression of *Fcgbp* and *Clca1*, encoding the intestinal Goblet-cell-secreted proteins IgGFc-binding protein (FCGBP) and calcium-activated chloride channel regulator 1 (CLCA1) [48, 49], was elevated in the colon of feralized and co-housed mice. In contrast, the genes encoding the major mucus glycoproteins (*Muc2*, Mucin 2, *Muc3*, Mucin 3 [63]) remained largely unaffected. While both FCGBP and CLCA1 are central components of the intestinal mucus, their functional roles remain incompletely understood. We have previously reported increased expression of these genes following feralization [20], but did not observe changes in colonic or ileal mucus thickness or penetrability in feralized mice compared to conventionally reared lab mice. However, the elevated expression of these two genes in colonic IECs observed in this current study supports their involvement in organizing and maintaining the mucosal barrier following feralization and/or co-housing.

Both feralized and co-housed mice displayed an increased expression of *Ang4*, encoding the bacterial-membrane integrity-disrupting antimicrobial peptide Angiogenin-4 (Ang4) [46]. Ang4 is known to be expressed by Paneth cells and goblet cells [64–67]. Colonic Ang4-expression has more recently been attributed to a goblet cell subpopulation called Reg4-expressing deep crypt secretory cells, which are proposed to function as the Paneth cell of the colon [68, 69]. Ang4 expression is altered in response to changes in the microbiota and suggested to promote the growth of beneficial bacteria, reducing the abundance of potential pathogens [46]. Thus, the increased expression of *Ang4* observed in both feralized and co-housed mice may contribute to restoring microbial homeostasis by adjusting the distribution of the environmentally acquired microbes, which potentially include pathogens from wild mice [21, 24].

Increased expression of *Retnlb*, encoding Resistin-like beta (RELMβ), was also a feature of feralized and co-housed mice. RELMβ is a lytic pore-forming bactericidal protein [45] expressed in response to increased microbial exposure, displaying elevated levels in conventionally reared mice vs GF mice in both Paneth cells and colonic mucosa [67, 70]. As for Ang4, RELMβ has recently been linked to expression in Reg4-expressing deep crypt secretory cells, further supporting that this goblet cell subtype acts as a source of antimicrobials in the colon [71]. The colon tissue-associated microbiota of *Retnlb*-gene-deficient mice has shown elevated levels of Pseudomonadota, attributed to *Helicobacteracaea* [45] and thus the enrichment of *Helicobacter* spp. in the colonic mucosal-associated microbiota of feralized and co-housed mice in our study aligns with elevated expression of *Retlnb*.

Respectively, *Reg3g* and *Reg3b* encode Regenerating Islet-Derived Protein 3 gamma (Reg3g) and beta (Reg3b), peptidoglycan-binding proteins that kill bacteria through lytic membrane-pore formation [47, 72]. As *Reg3* expression is normally induced via microbial sensing by toll-like receptors or IL-22 signaling, and reduced after microbial depletion, the reduced colonic expression of both *Reg3* genes we observed in feralized and co-housed mice seems counterintuitive. Yet, a recent study revealed detrimental effects of REG3γ-overproduction on intestinal inflammation by hindering growth of protective commensals [73]. Thus, the decreased mRNA-levels of *Reg3b* and *Reg3g* in co-housed animals may play a role in preventing the loss of beneficial commensals.

Z-DNA-binding protein 1 (ZBP1), encoded by *Zbp1*, induces inflammation and cell death in response to sensing Z-form nucleic acids that arise during viral infection [50]. Enhanced expression of *Zbp1* following feralization and co-housing could coincide with the transmission of viruses in co-housed mice reported previously [24]. Moreover, we observed increased expression of *Gsdmc*, encoding Gasdermin-C (GSDMC), in the naturalized mouse colon. GSDMC appears to be critical for helminth expulsion [51, 74]. Helminth parasites may alter the composition of the gut microbiota, as shown in wild-caught mice [75] and are well known to significantly change both local and systemic immunity [76]. However, while parasitic eggs/cysts were detected in some of the wild and co-housed mice and one single feralized mouse, the current data set is too small to allow conclusions regarding any potential parasitic influence.

Besides antimicrobial peptides, ROS are central in maintaining a healthy distance between gut microbes and the epithelium. Dual oxidase 2 (DUOX2) encoded by *Duox2*, produces hydrogen peroxide, killing encroaching bacteria [11]. Microbial enrichment by feralization markedly induced elevated expression of *Duox2* in the colon, while only wild-mouse microbes affected expression levels in the small intestine. Colonic and small intestinal expression of *Duox2* is regulated by different signaling pathways, and the composition and density of the gut microbiota in the colon, possibly explaining these discrepancies [11]. All taken together, the transfer of mouse-specific microorganisms from wild mice to lab mice during co-housing appears to facilitate the most robust changes in gene expression observed in the naturalized mice in this study.

While our study highlights how the different naturalization approaches affect laboratory mouse microbiota and immune components differently, limitations to these research models should be noted. The wild or pet-store mouse microbiotas will be different depending on where they are collected [77, 78], but to some extent, this also applies to laboratory mice whose microbiotas differ between facilities [79]. The farmyard-like habitat in which mice are feralized includes a wide array of poorly controllable environmental conditions, such as nutritional elements, odors, and other factors that have been frequently discussed in previous studies of naturalized mice [17]. However, the objective of naturalization models is to study mice in the context of a natural complex microbiota and experienced immune system, and these uncontrolled conditions are intrinsic components of the natural world, including the diverse lives of humans, who are usually the targets for preclinical studies.

The choice of naturalization method will largely depend on study aim, context, and resources, as has been discussed in a recently published perspective [3]. The feralization model provides flexibility in choosing which factors to introduce, as well as the timing of introduction, offering opportunities to study dynamics of host interactions in various diverse environments. Co-housing experiments present further challenges, as wild or pet shop-purchased mice may not be easily or timely available, and pose challenges in terms of maintenance, consistency, as well as ethical and animal welfare considerations. The data presented herein along with previous studies [19–21, 24] suggest that feralization induces changes in the intestinal microbiome, barrier functions, and regional mucosal immune parameters of relevance to local conditions in the colon. Meanwhile co-housing with wild or pet-store mice provides transfer of mouse-adapted microbes, including viral pathogens, fostering a robust immune experience on a more systemic level [23].

In conclusion, this study elucidates how various types of naturalization of laboratory mice distinctly influence immune status and gut microbiota profiles in a cross comparison. Co-housing with wild mice appears to elicit stronger effects on gene expression and proportion of immune cells, suggesting that transfer of microbes between mice is more potent in enhancing recipient’s immune responses than environmental stimuli alone. We identify microbes likely derived from different sources in the naturalization setups, which may provide researchers with a narrowed-down list of biologically relevant candidates for use in more reductionistic studies on microbial–host interactions. Identifying the species through methods such as shotgun metagenome sequencing will enhance the understanding of their role in naturalization models. The comparison of naturalization models presented herein, and their effects on host responses will help to guide refined use of these models in future studies involving naturalized mice, where the impact of these effects and the relevance to experimental outcomes should be considered.

## Supplementary Material

kyaf002_suppl_Supplementary_Tables_S1-S5

kyaf002_suppl_Supplementary_Figures_S1-S6

## Data Availability

The authors confirm that the data supporting the findings of this study are openly available within the article, its Supplementary Materials and/or upon reasonable request to the corresponding author. Metagenomic sequences are deposited to NCBIs Sequence Read Archive (https://www.ncbi.nlm.nih.gov/sra) under the accession number PRJNA1116057.

## Abbreviations

Ang4: angiogenin-4
B6: C57BL/6JRj
CLCA1: calcium-activated chloride channel regulator 1
Cq: quantification cycle
DUOX2: dual oxidase 2
FCGBP: IgGFc-binding protein
GSDMC: gasdermin-C
IEC: intestinal epithelial cell
IFAT: immunofluorescence antibody test
IFC: integrated fluidic circuit
IFNγ: interferon gamma
mLN: mesenteric lymph node
NMDS: non-metric multi-dimensional scaling
Reg3g: regenerating islet-derived protein 3 gamma
Reg3b: regenerating islet-derived protein 3 beta
RELMβ: resistin-like beta
SPF: specific pathogen free
Trm: tissue-resident memory T cell
ZBP1: Z-DNA-binding protein 1
zOTU: zero-radiance operational taxonomic unit

## References

[1] McFall-Ngai M , Hadfield MG, Bosch TCG, Carey HV, Domazet-Lošo T, Douglas AE, et al Animals in a bacterial world, a new imperative for the life sciences. Proc Natl Acad Sci USA 2013, 110, 3229–36. doi: https://doi.org/10.1073/pnas.1218525110. 23391737 PMC3587249

[2] Masopust D , Sivula CP, Jameson SC. Of mice, dirty mice, and men: using mice to understand human immunology. J Immunol 2017, 199, 383–8. doi: https://doi.org/10.4049/jimmunol.1700453. 28696328 PMC5512602

[3] Rehermann B , Graham AL, Masopust D, Hamilton SE. Integrating natural commensals and pathogens into preclinical mouse models. Nat Rev Immunol 2024. doi: https://doi.org/10.1038/s41577-024-01108-3. PMC12126596 39562646

[4] Donaldson GP , Lee SM, Mazmanian SK. Gut biogeography of the bacterial microbiota. Nat Rev Microbiol 2016, 14, 20–32. doi: https://doi.org/10.1038/nrmicro3552. 26499895 PMC4837114

[5] Belkaid Y , Harrison OJ. Homeostatic immunity and the microbiota. Immunity 2017, 46, 562–76. doi: https://doi.org/10.1016/j.immuni.2017.04.008. 28423337 PMC5604871

[6] Chelakkot C , Ghim J, Ryu SH. Mechanisms regulating intestinal barrier integrity and its pathological implications. Exp Mol Med 2018, 50, 1–9. doi: https://doi.org/10.1038/s12276-018-0126-x. PMC6095905 30115904

[7] Eshleman EM , Alenghat T. Epithelial sensing of microbiota-derived signals. Genes Immun 2021, 22, 237–46. doi: https://doi.org/10.1038/s41435-021-00124-w. 33824498 PMC8492766

[8] Matziouridou C , Rocha SDC, Haabeth OA, Rudi K, Carlsen H, Kielland A. iNOS- and NOX1-dependent ROS production maintains bacterial homeostasis in the ileum of mice. Mucosal Immunol 2018, 11, 774–84. doi: https://doi.org/10.1038/mi.2017.106. 29210363

[9] Ra YE , Bang Y-J. Balancing act of the intestinal antimicrobial proteins on gut microbiota and health. J Microbiol 2024, 62, 167–79. doi: https://doi.org/10.1007/s12275-024-00122-3. 38630349 PMC11090926

[10] Soderholm AT , Pedicord VA. Intestinal epithelial cells: at the interface of the microbiota and mucosal immunity. Immunology 2019, 158, 267–80. doi: https://doi.org/10.1111/imm.13117. 31509239 PMC6856932

[11] Sommer F , Bäckhed F. The gut microbiota engages different signaling pathways to induce Duox2 expression in the ileum and colon epithelium. Mucosal Immunol 2015, 8, 372–9. doi: https://doi.org/10.1038/mi.2014.74. 25160818

[12] Masopust D , Soerens AG. Tissue-resident T cells and other resident leukocytes. Annu Rev Immunol 2019, 37, 521–46. doi: https://doi.org/10.1146/annurev-immunol-042617-053214. 30726153 PMC7175802

[13] Abolins S , King EC, Lazarou L, Weldon L, Hughes L, Drescher P, et al The comparative immunology of wild and laboratory mice, *Mus musculus domesticus*. Nat Commun 2017, 8, 14811. doi: https://doi.org/10.1038/ncomms14811. 28466840 PMC5418598

[14] Abolins S , Lazarou L, Weldon L, Hughes L, King EC, Drescher P, et al The ecology of immune state in a wild mammal, *Mus musculus domesticus*. PLoS Biol 2018, 16, e2003538–38-e. doi: https://doi.org/10.1371/journal.pbio.2003538. 29652925 PMC5919074

[15] Boysen P , Eide DM, Storset AK. Natural killer cells in free-living *Mus musculus* have a primed phenotype. Mol Ecol 2011, 20, 5103–10. doi: https://doi.org/10.1111/j.1365-294X.2011.05269.x. 21895821

[16] Weldon L , Abolins S, Lenzi L, Bourne C, Riley EM, Viney M. The gut microbiota of wild mice. PLoS One 2015, 10, e0134643. doi: https://doi.org/10.1371/journal.pone.0134643. 26258484 PMC4530874

[17] Graham AL. Naturalizing mouse models for immunology. Nat Immunol 2021, 22, 111–7. doi: https://doi.org/10.1038/s41590-020-00857-2. 33495644

[18] Liu Q , Pickett T, Hodge D, Rios C, Arnold M, Dong G, et al Leveraging dirty mice that have microbial exposure to improve preclinical models of human immune status and disease. Nat Immunol 2024, 25, 947–50. doi: https://doi.org/10.1038/s41590-024-01842-9. 38750319 PMC11656454

[19] Arnesen H , Hitch TCA, Steppeler C, Müller MHB, Knutsen LE, Gunnes G, et al Naturalizing laboratory mice by housing in a farmyard-type habitat confers protection against colorectal carcinogenesis. Gut Microbes 2021, 13, 1993581. doi: https://doi.org/10.1080/19490976.2021.1993581. 34751603 PMC8583187

[20] Arnesen H , Markussen T, Birchenough G, Birkeland S, Nyström EEL, Hansson GC, et al Microbial experience through housing in a farmyard-type environment alters intestinal barrier properties in mouse colons. Sci Rep 2023, 13, 13701. doi: https://doi.org/10.1038/s41598-023-40640-5. 37607995 PMC10444815

[21] Arnesen H , Knutsen LE, Hognestad BW, Johansen GM, Bemark M, Pabst O, et al A model system for feralizing laboratory mice in large farmyard-like pens. Front Microbiol 2021, 11, 615661. doi: https://doi.org/10.3389/fmicb.2020.615661. 33505381 PMC7830425

[22] Rosshart SP , Herz J, Vassallo BG, Hunter A, Wall MK, Badger JH, et al Laboratory mice born to wild mice have natural microbiota and model human immune responses. Science 2019, 365, eaaw4361. doi: https://doi.org/10.1126/science.aaw4361. 31371577 PMC7377314

[23] Beura LK , Hamilton SE, Bi K, Schenkel JM, Odumade OA, Casey KA, et al Normalizing the environment recapitulates adult human immune traits in laboratory mice. Nature 2016, 532, 512–6. doi: https://doi.org/10.1038/nature17655. 27096360 PMC4871315

[24] Sanders AE , Arnesen H, Shepherd FK, Putri DS, Fiege JK, Pierson MJ, et al Comparison of mouse models of microbial experience reveals differences in microbial diversity and response to vaccination. mSphere 2024, 9, e0065423. doi: https://doi.org/10.1128/msphere.00654-23. 38286428 PMC10900878

[25] Reitmeier S , Kiessling S, Neuhaus K, Haller D. Comparing circadian rhythmicity in the human gut microbiome. STAR Protoc 2020, 1, 100148. doi: https://doi.org/10.1016/j.xpro.2020.100148. 33377042 PMC7757335

[26] Lagkouvardos I , Joseph D, Kapfhammer M, Giritli S, Horn M, Haller D, et al IMNGS: a comprehensive open resource of processed 16S rRNA microbial profiles for ecology and diversity studies. Sci Rep 2016, 6, 33721. doi: https://doi.org/10.1038/srep33721. 27659943 PMC5034312

[27] Edgar RC. Search and clustering orders of magnitude faster than BLAST. Bioinformatics 2010, 26, 2460–1. doi: https://doi.org/10.1093/bioinformatics/btq461. 20709691

[28] Kopylova E , Noé L, Touzet H. SortMeRNA: fast and accurate filtering of ribosomal RNAs in metatranscriptomic data. Bioinformatics 2012, 28, 3211–7. doi: https://doi.org/10.1093/bioinformatics/bts611. 23071270

[29] Pruesse E , Peplies J, Glöckner FO. SINA: accurate high-throughput multiple sequence alignment of ribosomal RNA genes. Bioinformatics 2012, 28, 1823–9. doi: https://doi.org/10.1093/bioinformatics/bts252. 22556368 PMC3389763

[30] Simonsen M , Mailund T, Pedersen CNS (eds). Rapid Neighbour-Joining. Berlin, Heidelberg: Springer Berlin Heidelberg, 2008. doi:10.1007/978-3-540-87361-7_10.

[31] Parte AC , Sardà Carbasse J, Meier-Kolthoff JP, Reimer LC, Göker M. List of Prokaryotic names with Standing in Nomenclature (LPSN) moves to the DSMZ. Int J Syst Evol Microbiol 2020, 70, 5607–12. doi: https://doi.org/10.1099/ijsem.0.004332. 32701423 PMC7723251

[32] Goodyear AW , Kumar A, Dow S, Ryan EP. Optimization of murine small intestine leukocyte isolation for global immune phenotype analysis. J Immunol Methods 2014, 405, 97–108. doi: https://doi.org/10.1016/j.jim.2014.01.014. 24508527

[33] Maniatis T , Sambrook J, Fritsch EF. Molecular Cloning: A Laboratory Manual. 2 edn. Cold Spring Harbor Laboratory, 1989. ISBN: 0-87969-309-6.

[34] Riccio C. RNA re-precipitation protocol V.2 2019. Retrieved from https://www.protocols.io/view/rna-re-precipitation-protocol-5qpvon11dl4o/v2?step=1

[35] Ruijter JM , van der Velden S, Ilgun A. LinRegPCR (11.0) Analysis of quantitative RTPCR data. Retrieved from https://www.gene-quantification.de/LinRegPCR_help_manual_v11.0.pdf

[36] R Core Team. R: A Language and Environment for Statistical Computing. Vienna, Austria: R Foundation for Statistical Computing, 2022.

[37] Lagkouvardos I , Fischer S, Kumar N, Clavel T. Rhea: a transparent and modular R pipeline for microbial profiling based on 16S rRNA gene amplicons. PeerJ 2017, 5, e2836. doi: https://doi.org/10.7717/peerj.2836. 28097056 PMC5234437

[38] Cáceres MD , Legendre P. Associations between species and groups of sites: indices and statistical inference. Ecology 2009, 90, 3566–74. doi: https://doi.org/10.1890/08-1823.1. 20120823

[39] Dufrene M , Legendre P. Species assemblages and indicator species: the need for a flexible asymmetrical approach. Ecol Monogr 1997, 67, 345–66. doi: https://doi.org/10.2307/2963459.

[40] Yoon SH , Ha SM, Kwon S, Lim J, Kim Y, Seo H, et al Introducing EzBioCloud: a taxonomically united database of 16S rRNA gene sequences and whole-genome assemblies. Int J Syst Evol Microbiol 2017, 67, 1613–7. doi: https://doi.org/10.1099/ijsem.0.001755. 28005526 PMC5563544

[41] Hulsen T , de Vlieg J, Alkema W. BioVenn—a web application for the comparison and visualization of biological lists using area-proportional Venn diagrams. BMC Genomics 2008, 9, 488. doi: https://doi.org/10.1186/1471-2164-9-488. 18925949 PMC2584113

[42] Livak KJ , Schmittgen TD. Analysis of relative gene expression data using real-time quantitative PCR and the 2−ΔΔCT method. Methods 2001, 25, 402–8. doi: https://doi.org/10.1006/meth.2001.1262. 11846609

[43] Kok L , Masopust D, Schumacher TN. The precursors of CD8+ tissue resident memory T cells: from lymphoid organs to infected tissues. Nat Rev Immunol 2022, 22, 283–93. doi: https://doi.org/10.1038/s41577-021-00590-3. 34480118 PMC8415193

[44] Piedra-Quintero ZL , Wilson Z, Nava P, Guerau-de-Arellano M. CD38: an immunomodulatory molecule in inflammation and autoimmunity. Front Immunol 2020, 11, 597959. doi: https://doi.org/10.3389/fimmu.2020.597959. 33329591 PMC7734206

[45] Propheter DC , Chara AL, Harris TA, Ruhn KA, Hooper LV. Resistin-like molecule β is a bactericidal protein that promotes spatial segregation of the microbiota and the colonic epithelium. Proc Natl Acad Sci USA 2017, 114, 11027–33. doi: https://doi.org/10.1073/pnas.1711395114. 28973871 PMC5651776

[46] Sultana MF , Suzuki M, Yamasaki F, Kubota W, Takahashi K, Abo H, et al Identification of crucial amino acid residues for antimicrobial activity of angiogenin 4 and its modulation of gut microbiota in mice. Front Microbiol 2022, 13, 900948. doi: https://doi.org/10.3389/fmicb.2022.900948. 35733962 PMC9207454

[47] Miki T , Okada N, Hardt W-D. Inflammatory bactericidal lectin RegIIIβ: friend or foe for the host? Gut Microbes 2018, 9, 179–87. doi: https://doi.org/10.1080/19490976.2017.1387344. 28985140 PMC5989794

[48] Ehrencrona E , van der Post S, Gallego P, Recktenwald CV, Rodriguez-Pineiro AM, Garcia-Bonete MJ, et al The IgGFc-binding protein FCGBP is secreted with all GDPH sequences cleaved but maintained by interfragment disulfide bonds. J Biol Chem 2021, 297, 100871. doi: https://doi.org/10.1016/j.jbc.2021.100871. 34126068 PMC8267560

[49] Nyström EEL , Arike L, Ehrencrona E, Hansson GC, Johansson MEV. Calcium-activated chloride channel regulator 1 (CLCA1) forms non-covalent oligomers in colonic mucus and has mucin 2-processing properties. J Biol Chem 2019, 294, 17075–89. doi: https://doi.org/10.1074/jbc.RA119.009940. 31570526 PMC6851300

[50] Zhi-Yu C , Puqi W, Hao L, Yu-Ze X, Bo-Xin Z, Cai-Ling H, et al A ZBP1 isoform blocks ZBP1-mediated cell death. Cell Reports 2024, 43, 114221. doi:10.1016/j.celrep.2024.114221. 38748877

[51] Lin Z , Chen Q, Ruan HB. To die or not to die: gasdermins in intestinal health and disease. Semin Immunol 2024, 71, 101865. doi: https://doi.org/10.1016/j.smim.2024.101865. 38232665 PMC10872225

[52] Abell GCJ , Cooke CM, Bennett CN, Conlon MA, McOrist AL. Phylotypes related to *Ruminococcus bromii* are abundant in the large bowel of humans and increase in response to a diet high in resistant starch. FEMS Microbiol Ecol 2008, 66, 505–15. doi: https://doi.org/10.1111/j.1574-6941.2008.00527.x. 18616586

[53] Mukherjee A , Lordan C, Ross RP, Cotter PD. Gut microbes from the phylogenetically diverse genus Eubacterium and their various contributions to gut health. Gut Microbes 2020, 12, 1802866. doi: https://doi.org/10.1080/19490976.2020.1802866. 32835590 PMC7524325

[54] Nagao-Kitamoto H , Leslie JL, Kitamoto S, Jin C, Thomsson KA, Gillilland MG 3rd, et al Interleukin-22-mediated host glycosylation prevents Clostridioides difficile infection by modulating the metabolic activity of the gut microbiota. Nat Med 2020, 26, 608–17. doi: https://doi.org/10.1038/s41591-020-0764-0. 32066975 PMC7160049

[55] Watanabe Y , Nagai F, Morotomi M. Characterization *of Phascolarctobacterium succinatutens* sp. nov., an Asaccharolytic, Succinate-utilizing bacterium isolated from human feces. Appl Environ Microbiol 2012, 78, 511–8. doi: https://doi.org/10.1128/AEM.06035-11. 22081579 PMC3255759

[56] Buddhasiri S , Sukjoi C, Kaewsakhorn T, Nambunmee K, Nakphaichit M, Nitisinprasert S, et al Anti-inflammatory effect of probiotic *Limosilactobacillus reuteri* KUB-AC5 against *Salmonella* infection in a mouse colitis model. Front Microbiol 2021, 12, 716761. doi: https://doi.org/10.3389/fmicb.2021.716761. 34497597 PMC8419263

[57] Li S , Wang C, Zhang C, Luo Y, Cheng Q, Yu L, et al Evaluation of the effects of different *Bacteroides vulgatus* strains against DSS-induced colitis. J Immunol Res 2021, 2021, 9117805. doi: https://doi.org/10.1155/2021/9117805. 34195297 PMC8181088

[58] Rosshart SP , Vassallo BG, Angeletti D, Hutchinson DS, Morgan AP, Takeda K, et al Wild mouse gut microbiota promotes host fitness and improves disease resistance. Cell 2017, 171, 1015–28.e13. doi: https://doi.org/10.1016/j.cell.2017.09.016. 29056339 PMC6887100

[59] Zhao B , Osbelt L, Lesker TR, Wende M, Galvez EJC, Hönicke L, et al *Helicobacter* spp. are prevalent in wild mice and protect from lethal *Citrobacter rodentium* infection in the absence of adaptive immunity. Cell Reports 2023, 42, 112549. doi: https://doi.org/10.1016/j.celrep.2023.112549. 37245209

[60] Overacre-Delgoffe AE , Bumgarner HJ, Cillo AR, Burr AHP, Tometich JT, Bhattacharjee A, et al Microbiota-specific T follicular helper cells drive tertiary lymphoid structures and anti-tumor immunity against colorectal cancer. Immunity 2021, 54, 2812–24.e4. doi: https://doi.org/10.1016/j.immuni.2021.11.003. 34861182 PMC8865366

[61] Labuda JC , Fong KD, McSorley SJ. Cohousing with dirty mice increases the frequency of memory T cells and has variable effects on intracellular bacterial infection. Immunohorizons 2022, 6, 184–90. doi: https://doi.org/10.4049/immunohorizons.2100069. 35210292 PMC9624231

[62] Kitakaze M , Uemura M, Hara T, Chijimatsu R, Motooka D, Hirai T, et al Cancer-specific tissue-resident memory T-cells express ZNF683 in colorectal cancer. Br J Cancer 2023, 128, 1828–37. doi: https://doi.org/10.1038/s41416-023-02202-4. 36869093 PMC10147592

[63] Hansson GC. Mucins and the microbiome. Annu Rev Biochem 2020, 89, 769–93. doi: https://doi.org/10.1146/annurev-biochem-011520-105053. 32243763 PMC8442341

[64] Burger-van Paassen N , Loonen LM, Witte-Bouma J, Korteland-van Male AM, de Bruijn AC, van der Sluis M, et al Mucin Muc2 deficiency and weaning influences the expression of the innate defense genes Reg3β, Reg3γ and angiogenin-4. PLoS One 2012, 7, e38798. doi: https://doi.org/10.1371/journal.pone.0038798. 22723890 PMC3378615

[65] Forman RA , deSchoolmeester ML, Hurst RJ, Wright SH, Pemberton AD, Else KJ. The goblet cell is the cellular source of the anti-microbial angiogenin 4 in the large intestine post *Trichuris muris* infection. PLoS One 2012, 7, e42248. doi: https://doi.org/10.1371/journal.pone.0042248. 22970115 PMC3435386

[66] Hooper LV , Stappenbeck TS, Hong CV, Gordon JI. Angiogenins: a new class of microbicidal proteins involved in innate immunity. Nat Immunol 2003, 4, 269–73. doi: https://doi.org/10.1038/ni888. 12548285

[67] Levy M , Thaiss CA, Zeevi D, Dohnalová L, Zilberman-Schapira G, Mahdi JA, et al Microbiota-modulated metabolites shape the intestinal microenvironment by regulating NLRP6 inflammasome signaling. Cell 2015, 163, 1428–43. doi: https://doi.org/10.1016/j.cell.2015.10.048. 26638072 PMC5665753

[68] Abo H , Sultana MF, Kawashima H. Dual function of angiogenin-4 inducing intestinal stem cells and apoptosis. Front Cell Dev Biol 2023, 11, 1181145. doi: https://doi.org/10.3389/fcell.2023.1181145. 38020881 PMC10651741

[69] Sasaki N , Sachs N, Wiebrands K, Ellenbroek SIJ, Fumagalli A, Lyubimova A, et al Reg4+ deep crypt secretory cells function as epithelial niche for Lgr5+ stem cells in colon. Proc Natl Acad Sci USA 2016, 113, E5399–407. doi: https://doi.org/10.1073/pnas.1607327113. 27573849 PMC5027439

[70] Reikvam DH , Erofeev A, Sandvik A, Grcic V, Jahnsen FL, Gaustad P, et al Depletion of murine intestinal microbiota: effects on gut mucosa and epithelial gene expression. PLoS One 2011, 6, e17996. doi: https://doi.org/10.1371/journal.pone.0017996. 21445311 PMC3061881

[71] Schumacher MA , Liu CY, Katada K, Thai MH, Hsieh JJ, Hansten BJ, et al Deep crypt secretory cell differentiation in the colonic epithelium is regulated by Sprouty2 and Interleukin 13. Cell Mol Gastroenterol Hepatol 2023, 15, 971–84. doi: https://doi.org/10.1016/j.jcmgh.2022.11.004. 36414210 PMC9982040

[72] Lehotzky RE , Partch CL, Mukherjee S, Cash HL, Goldman WE, Gardner KH, et al Molecular basis for peptidoglycan recognition by a bactericidal lectin. Proc Natl Acad Sci USA 2010, 107, 7722–7. doi: https://doi.org/10.1073/pnas.0909449107. 20382864 PMC2867859

[73] Kyung KJ , Thomas H, Mariya L, Yi D, Gregory P, Frank Y, et al Antimicrobial overproduction sustains intestinal inflammation by inhibiting Enterococcus colonization. Cell Host Microbe 2023, 31, 1450–68.e8. doi: 10.1016/j.chom.2023.08.002. 37652008 PMC10502928

[74] Ming Z , Kaiqun R, Xiwen X, Yue X, Yujie Z, Jason CM, et al Epithelial STAT6 O-GlcNAcylation drives a concerted anti-helminth alarmin response dependent on tuft cell hyperplasia and Gasdermin C. Immunity 2022, 55, 623–38.e5. doi:10.1016/j.immuni.2022.03.009. 35385697 PMC9109499

[75] Kreisinger J , Bastien G, Hauffe HC, Marchesi J, Perkins SE. Interactions between multiple helminths and the gut microbiota in wild rodents. Philos Trans R Soc B Biol Sci 2015, 370, 20140295. doi: https://doi.org/10.1098/rstb.2014.0295. PMC4528493 26150661

[76] Kasal DN , Warner LM, Bryant AS, Tait Wojno E, von Moltke J. Systemic immune modulation by gastrointestinal nematodes. Annu Rev Immunol 2024, 42, 259–88. doi: https://doi.org/10.1146/annurev-immunol-090222-101331. 38277692 PMC12153512

[77] Linnenbrink M , Wang J, Hardouin EA, Künzel S, Metzler D, Baines JF. The role of biogeography in shaping diversity of the intestinal microbiota in house mice. Mol Ecol 2013, 22, 1904–16. doi: https://doi.org/10.1111/mec.12206. 23398547

[78] Kreisinger J , Čížková D, Vohánka J, Piálek J. Gastrointestinal microbiota of wild and inbred individuals of two house mouse subspecies assessed using high-throughput parallel pyrosequencing. Mol Ecol 2014, 23, 5048–60. doi: https://doi.org/10.1111/mec.12909. 25204516

[79] Rausch P , Basic M, Batra A, Bischoff SC, Blaut M, Clavel T, et al Analysis of factors contributing to variation in the C57BL/6J fecal microbiota across German animal facilities. Int J Med Microbiol 2016, 306, 343–55. doi: https://doi.org/10.1016/j.ijmm.2016.03.004. 27053239

