# Supplementary material for: Comparison of naturalization mouse model setups uncover distinct effects on intestinal mucosa depending on microbial experience
Source: Discov Immunol. 2025 Feb 1;4(1):kyaf002. doi: 10.1093/discim/kyaf002 (PMC11892432; doi:10.1093/discim/kyaf002)
Supplement: kyaf002_suppl_Supplementary_Tables_S1-S5 [file kyaf002_suppl_supplementary_tables_s1-s5.zip › Supplementary Tables S1 to S5/Supplementary Tables S1 to S5.docx]

# Supplementary material

## Supplementary Tables and Table Captions

Supplementary Table S1**: Details of antibodies used in immunophenotyping.**

| **Antibody** | **Fluorochrome** | **Clone** | **Vendor** |
| --- | --- | --- | --- |
| B220 | PE | RA3-6B2 | Tonbo |
| CD103 | BV510 | 2E7 | Biolegend |
| CD11b | Alexa700 | M1/70 | BD |
| CD3 | APC-e780 | 17A2 | Thermo Fischer Scientific |
| CD38 | VioBlue | 90/CD38 | Miltenyi Biotech |
| CD4 | BV650 | RM4-5 | Biolegend |
| CD4 | PE-Dazzle594 | GK1.5 | Biolegend |
| CD44 | BV786 | IM7 | Biolegend |
| CD45 | BV594 | 30F11 | Thermo Fischer Scientific |
| CD5 | BV510 | 53-7.3 | BD |
| CD62L | BV605 | MEL14 | Biolegend |
| CD69 | BV421 | H1.2F3 | Biolegend |
| CD8α | Alexa700 | 53-6.7 | Thermo Fischer Scientific |
| CD8β | PerCP-Cy5.5 | YTS156.7.7 | Biolegend |
| Foxp3 | PE | FJK-16s | Thermo Fischer Scientific |
| IFN- γ | PE | XMG1.2 | Thermo Fischer Scientific |
| NK1.1 | efluor450 | PK136 | Thermo Fischer Scientific |
| TCR-β | APC | H57-597 | Tonbo |
| TCR-γδ | FITC | GL3 | BD |

Supplementary Table S2**: RT-qPCR primers.** Full name and 5’ – 3’ sequence.

| **Full target gene name** | **Primer name** | **5’ – 3’ sequence** |
| --- | --- | --- |
| Alkaline phosphatase, intestinal | Alpi-Forward | TCGCCACTCAACTCATCTCC |
|  | Alpi-Reverse | AGTCCCCTTGGGAAACATGAA |
| Angiogenin, ribonuclease A family, member 4 | Ang4-Forward | CTCCAGGAGCACACAGCTA |
|  | Ang4-Reverse | CAGCACGAAGACCAACAACA |
| Cadherin 1 | Cdh1-Forward | ATTGCAAGTTCCTGCCATCC |
|  | Cdh1-Reverse | CAGTAGGAGCAGCAGGATCA |
| Chloride channel accessory 1 | Clca1-Forward | ACAACCACTAAGGTGGCCTA |
|  | Clca1-Reverse | GAGCTCGCTTGAATGCTGTA |
| Defensin, alpha, 3 | Defa3-Forward | CCCAGAAGGCTCTTCTCTTCA |
|  | Defa3-Reverse | CTTTCTGCAGGTCCCATTCA |
| Defensin, alpha, 24 | Defa24-Forward | CAGAAGGCGCTTCTCTTCAA |
|  | Defa24-Reverse | TTTGCAGCCTCTTGCTCTAC |
| Dual oxidase 2 | Duox2-Forward | GGCAGCCAGATGCTCTGTAA |
|  | Duox2-Reverse | ATGTCAGCCAGCCACTCAAA |
| F11 receptor | F11r-Forward | TGGAGTGGAAGTTCGTCCAA |
|  | F11r-Reverse | AGGTGACTCGGTCCGCATA |
| Fc fragment of IgG binding protein | Fcgbp-Forward | ATCGAGCAATGTGGCTGCTA |
|  | Fcgbp-Reverse | CAATGCTGCTGGCAGTTTTCA |
| Glyceraldehyde-3-phosphate dehydrogenase | Gapdh-Forward | CAAGGTCATCCCAGAGCTGAA |
|  | Gapdh-Reverse | CAGATCCACGACGGACACA |
| Gasdermin C | Gsdmc-Forward | AGGTTCAGAGTAAGAGCATCCC |
|  | Gsdmc-Reverse | ATGTGGGCAACTGATCCAAC |
| Gasdermin D | Gsdmd-Forward | GAGCCCAGTGCTCCAGAA |
|  | Gsdmd-Reverse | TGTTCCCATCGACGACATCA |
| Heme oxygenase 1 | Hmox1-Forward | TCAAGCACAGGGTGACAGAA |
|  | Hmox1-Reverse | ATCACCTGCAGCTCCTCAAA |
| Interleukin 10 | Il10-Forward | AAAGGACCAGCTGGACAACA |
|  | Il10-Reverse | TAAGGCTTGGCAACCCAAGTA |
| Interleukin 18 | Il18-Forward | CAAAGAAAGCCGCCTCAAAC |
|  | Il18-Reverse | GACGCAAGAGTCTTCTGACA |
| Interleukin 1 beta | Il1b-Forward | TGGCAACTGTTCCTGAACTCA |
|  | Il1b-Reverse | GGGTCCGTCAACTTCAAAGAAC |
| Interleukin 25 | Il25-Forward | CTCTCTCAGAAGGCCTGTCA |
|  | Il25-Reverse | CCCACGATCATTGCCAAGAA |
| Intelectin 1 (galactofuranose binding) | Itln1-Forward | TCTTTTCCTCTCTGCCCAGAA |
|  | Itln1-Reverse | GTGCGCAGGAAATAGAGACC |
| Lipocalin 2 | Lcn2-Forward | GCTACAATGTCACCTCCATCC |
|  | Lcn2-Reverse | CCCTGGAGCTTGGAACAAA |
| Mucin 2 | Muc2-Forward | CAGCACACCAACCAAAACCA |
|  | Muc2-Reverse | CACAGCCACCAGGTCTCATTA |
| Mucin 3, intestinal | Muc3-Forward | CCGGAGTATGAAGGGGTTATCA |
|  | Muc3-Reverse | ACTTGGCCTTCAGGATGACA |
| Myosin, light polypeptide kinase | Mylk-Forward | TTCAACAGGGTCACCAACCA |
|  | Mylk-Reverse | TCCAGGAAAGCTTGGGAGAC |
| NLR family, pyrin domain containing 6 | Nlrp6-Forward | CACCTCGGTGCTTCTCTCC |
|  | Nlrp6-Reverse | TTCACCTTAGCATGCTGTCGTA |
| Nucleotide-binding oligomerization domain containing 1 | Nod1-Forward | GTGGCTTTGGCTGTGAAGAA |
|  | Nod1-Reverse | TTTGCCCCTTCGTCTCCAA |
| Nucleotide-binding oligomerization domain containing 2 | Nod2-Forward | AAGCCCTGGCTGAAGTTGTA |
|  | Nod2-Reverse | CATGCTGCCAATGTTGTTTCC |
| Nitric oxide synthase 2, inducible | Nos2-Forward | GAGGAGCAGGTGGAAGACTA |
|  | Nos2-Reverse | GGAAAAGACTGCACCGAAGATA |
| NADPH oxidase 1 | Nox1-Forward | GTGCCGACAACAAGCTCAAA |
|  | Nox1-Reverse | GCAAAGGCACCTGTCTCTCTA |
| NAD(P)H dehydrogenase, quinone 1 | Nqo1-Forward | AAGCTGCAGACCTGGTGATA |
|  | Nqo1-Reverse | ACGAGCACTCTCTCAAACCA |
| Occludin | Ocln-Forward | GAATGGCAAGCGATCATACCC |
|  | Ocln-Reverse | GAATCTCCTGGGCCACTTCA |
| Regenerating islet-derived 3 beta | Reg3b-Forward | CTTTCTGTGGCAGCTTGTCA |
|  | Reg3b-Reverse | TAGGGCAACTTCACCTCACA |
| Regenerating islet-derived 3 gamma | Reg3g-Forward | GTATGGATTGGGCTCCATGAC |
|  | Reg3g-Reverse | CATCAGCATTGCTCCACTCC |
| Resistin like beta | Retnlb-Forward | CCTAAGACGATCTCCTGCACTA |
|  | Retnlb-Reverse | AGCACATCCAGTGACAACCA |
| Serum amyloid A1 | Saa1-Forward | ATCTCTCATGTGTGTATCCCACAA |
|  | Saa1-Reverse | TACCCTCTCCTCCTCAAGCA |
| TATA box binding protein | Tbp-Forward | ACCAGAACAACAGCCTTCCA |
|  | Tbp-Reverse | AAAGATGGGAATTCCAGGAGTCA |
| Tumor growth factor beta | Tgfb1-Forward | GCTGCGCTTGCAGAGATTAA |
|  | Tgfb1-Reverse | GTAACGCCAGGAATTGTTGCTA |
| Tight junction protein-1 | Tjp1-Forward | TCTGGCATCATTCGCCTTCA |
|  | Tjp1-Reverse | TCAACCGCATTTGGCGTTAC |
| Toll-like receptor 2 | Tlr2-Forward | TGCATCACCGGTCAGAAAAC |
|  | Tlr2-Reverse | AGCCAAAGAGCTCGTAGCA |
| Toll-like receptor 4 | Tlr4-Forward | GTTCTTCTCCTGCCTGACAC |
|  | Tlr4-Reverse | GCTGAGTTTCTGATCCATGCA |
| Toll-like receptor 5 | Tlr5-Forward | ATGGATGGATGCTGAGTTCCC |
|  | Tlr5-Reverse | CTGGCCATGAAGATCACACCTA |
| Z-DNA-binding protein 1 | Zbp1-Forward | TGGCAGAAGCTCCTGTTGAC |
|  | Zbp1-Reverse | CCAGCTGGCCAATCTTCACA |

Supplementary Table S3: Results from indicator species analyses (.xlsx file).

Supplementary Table S4**: Presence and grade of parasites in samples from the various experimental groups.** *Samples from individual mice; **each analyzed sample was pooled from 2-3 mice; +, 1-9; ++, 10-49, and +++, >50 visible parasite eggs at 4× or cysts/oocysts at 20×, respectively.

|  | **Wild mice co-housed with Fer-Co*** | | **Wild mice co-housed with Co*** | | **Fer-Co**** | | **Co** | | **Fer** | | **Lab** | |
| --- | --- | --- | --- | --- | --- | --- | --- | --- | --- | --- | --- | --- |
| Total n = | 5 | | 7 | | 6 | | 6 | | 5 | | 4 | |
|  | Presence | Grade | Presence | Grade | Presence | Grade | Presence | Grade | Presence | Grade | Presence | Grade |
| **Sucrose floatation** |  |  |  |  |  |  |  |  |  |  |  |  |
| *Heligmosomoides polygyrus* | 0 |  | 1 | + | 0 |  | 0 |  | 0 |  | 0 |  |
| *Eimeria* | 0 |  | 1 | + | 0 |  | 0 |  | 0 |  | 0 |  |
|  |  |  |  |  |  |  |  |  |  |  |  |  |
| **IFAT** |  |  |  |  |  |  |  |  |  |  |  |  |
| Cryptosporidium oocysts | 2 | + | 1 | + | 3 | + | 2 | + | 0 |  | 0 |  |
| Giardia cysts | 0 |  | 1 | + | 0 |  | 0 |  | 1 | + | 0 |  |

Supplementary Table S5: Gene expression data and results from statistical analyses (.xlsx file).
