# Supplementary material for: Comparison of naturalization mouse model setups uncover distinct effects on intestinal mucosa depending on microbial experience
Source: Discov Immunol. 2025 Feb 1;4(1):kyaf002. doi: 10.1093/discim/kyaf002 (PMC11892432; doi:10.1093/discim/kyaf002)
Supplement: kyaf002_suppl_Supplementary_Figures_S1-S6 [file kyaf002_suppl_supplementary_figures_s1-s6.zip › Supplementary Figures S1 to S5/Supplementary Figures S1 to S5.docx]

## Supplementary Figure Captions

Supplementary Figure S1. **Immunophenotyping gating strategies**. T cell subsets of single cell suspensions isolated from **(A**) mLNs and **(B)** colon/small intestine lamina propria, **(C)** expression of IFNγ on *ex vivo* stimulated T cells from single cell suspensions isolated from mesenteric lymph nodes, **(D)** B cell subsets in single cell suspensions isolated from intestines.

## **Supplementary Figure S2**. RNA integrity assessed by RNA 6000 Nano Assay on Agilent 2100 Bioanalyzer (Agilent Technologies).

Supplementary Figure S3. Discontinued feralization, i.e., moving feralized mice back to a laboratory setting, resulted in a microbiota composition middling that of laboratory and fully feralized mice. (A) Multi-dimensional scaling (MDS) plot of mucosa-associated microbiota profiles (generalized UniFrac distances) at endpoint (t2). Significance of separation was determined by PERMANOVA, and dispersion of groups determined by PERMDISP. d = distance scale. (B) Richness (observed number of OTUs) and Shannon effective counts. Box plots show median (line), mean (+), IQR (box) and minimum to maximum (whiskers). Letters designate significant (p ≤ 0.05) differences between groups at each timepoint determined by two-way ANOVA and Tukey’s multiple comparisons tests following significant ANOVAs. Absence of letters indicate no significant differences detected. (C) Taxonomic binning at phylum level, presented as relative abundance across groups at endpoint (t2). Lab (t0), n = 10; Lab (t2), n = 12; Fer-Lab, n = 6, Fer (t0), n = 10; Fer (t1), n = 6; Fer (t2), n = 11.

## Supplementary Figure S4. Mucosal-associated microbiota composition in mice jejunums significantly differed dependent on source of microbial exposure. (A) Multi-dimensional scaling (MDS) plot of mucosa-associated microbiota profiles (generalized UniFrac distances) at endpoint (t2). Significance of separation was determined by PERMANOVA, and dispersion of groups determined by PERMDISP. d = distance scale. (B) Richness (observed number of OTUs) and Shannon effective counts. Box plots show median (line), mean (+), IQR (box) and minimum to maximum (whiskers). Letters designate significant (p ≤ 0.05) differences between groups at each timepoint determined by two-way ANOVA and Tukey’s multiple comparisons tests following significant ANOVAs. (C) Taxonomic binning at phylum level, presented as relative abundance across groups at endpoint (t2). Lab, n = 7; Fer-Lab, n = 6; Fer, n = 5; Co, n = 7; Fer-Co, n = 8; Wild, n = 3.

Supplementary Figure S5. **Supplementary immunophenotyping data.** (**A**) Phenotypic markers of T cell subsets in small intestinal lamina propria. Lab, n = 12; Fer-Lab, n = 6; Fer, n = 12; Co, n = 12; Fer-Co, n = 12. (**B**) Phenotypic markers of B cell subsets in mesenteric lymph nodes. Lab, n = 12; Fer-Lab, n = 6; Fer, n = 12; Co, n = 12; Fer-Co, n = 12. (**C**) Cells from mesenteric lymph nodes were incubated with PMA/Ionomycin for 4 h and stained for immunophenotyping of intracytoplasmic IFNγ. Lab, n = 7; Fer-Lab, n = 4; Fer, n = 8; Co, n = 10; Fer-Co, n = 7. All graphs are presented as mean, with the standard deviation (SD) shown by whiskers. Different letters indicate statistical significance at alpha level 0.05 as determined by one-way ANOVA followed by Tukey’s multiple comparisons tests in cases of significant ANOVA, or non-parametric Kruskal-Wallis test followed by Dunn’s multiple comparisons tests in case of significant Kruskal-Wallis. Non-parametric statistical methods were applied to the graphs marked §.

## Supplementary Figure S6. Heatmap of gene expression levels in small intestine measured by high-throughput microfluidic RT-qPCR. Heatmap shows log2-fold change values for all measured genes, grouped according to experimental groups. General function is indicated by color coding: red, antimicrobial; purple, ROS/RNS; green, barrier/mucus; orange, inflammation; blue, immunosurveillance. Significant differences were determined by either one-way ANOVA, Kruskal-Wallis test, or Brown-Forsythe test, followed by Tukey’s, Dunn’s, or Dunnett’s T3 multiple comparisons tests in cases of significant ANOVA, respectively (see Supplementary Table S5).
