# Supplementary figures and images for: Comparison of naturalization mouse model setups uncover distinct effects on intestinal mucosa depending on microbial experience
Source: Discov Immunol. 2025 Feb 1;4(1):kyaf002. doi: 10.1093/discim/kyaf002 (PMC11892432; doi:10.1093/discim/kyaf002)

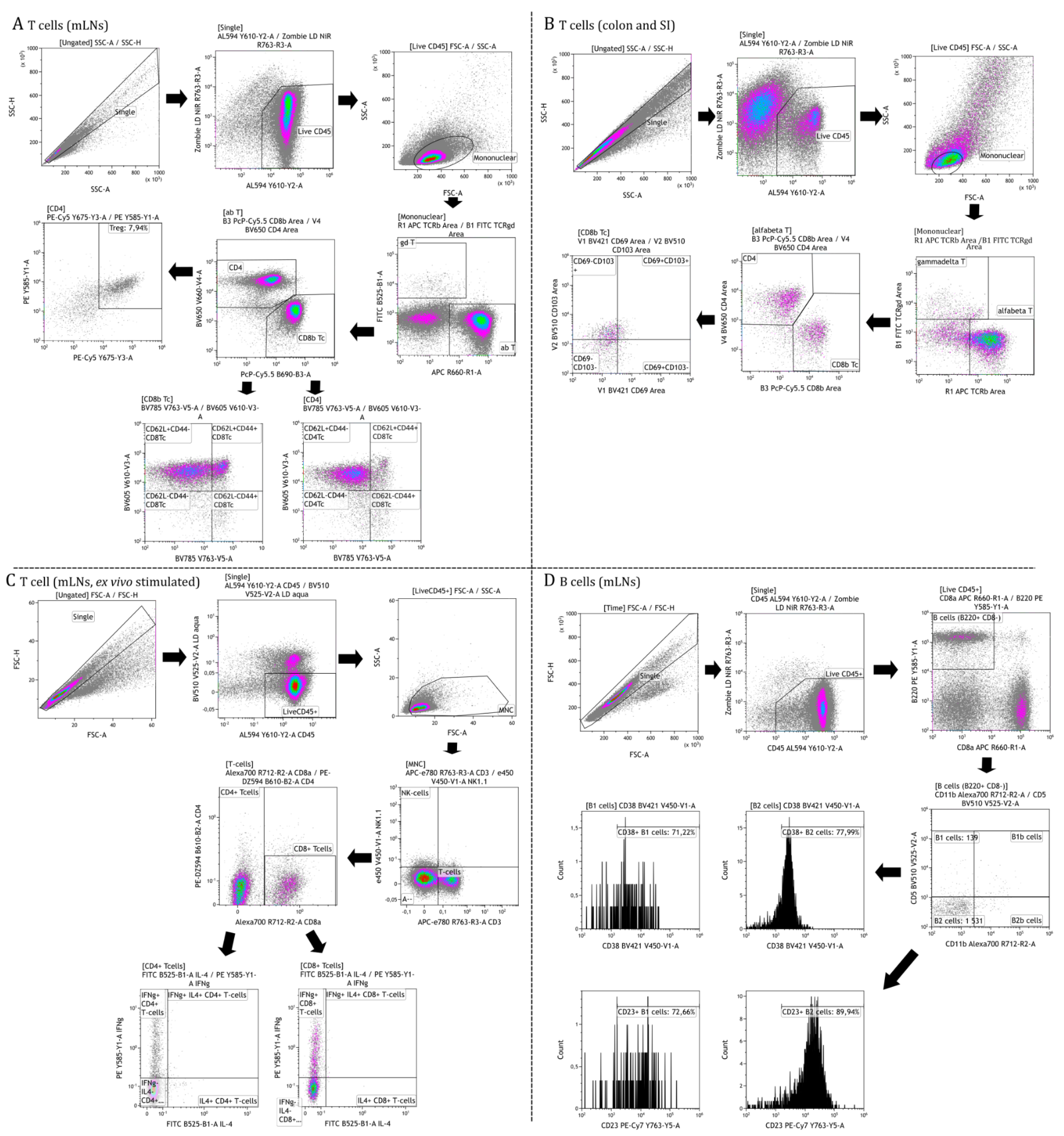

Supplement: kyaf002_suppl_Supplementary_Figures_S1-S6 [file kyaf002_suppl_supplementary_figures_s1-s6.zip › Supplementary Figures S1 to S5/Supplementary Figure S1.png]

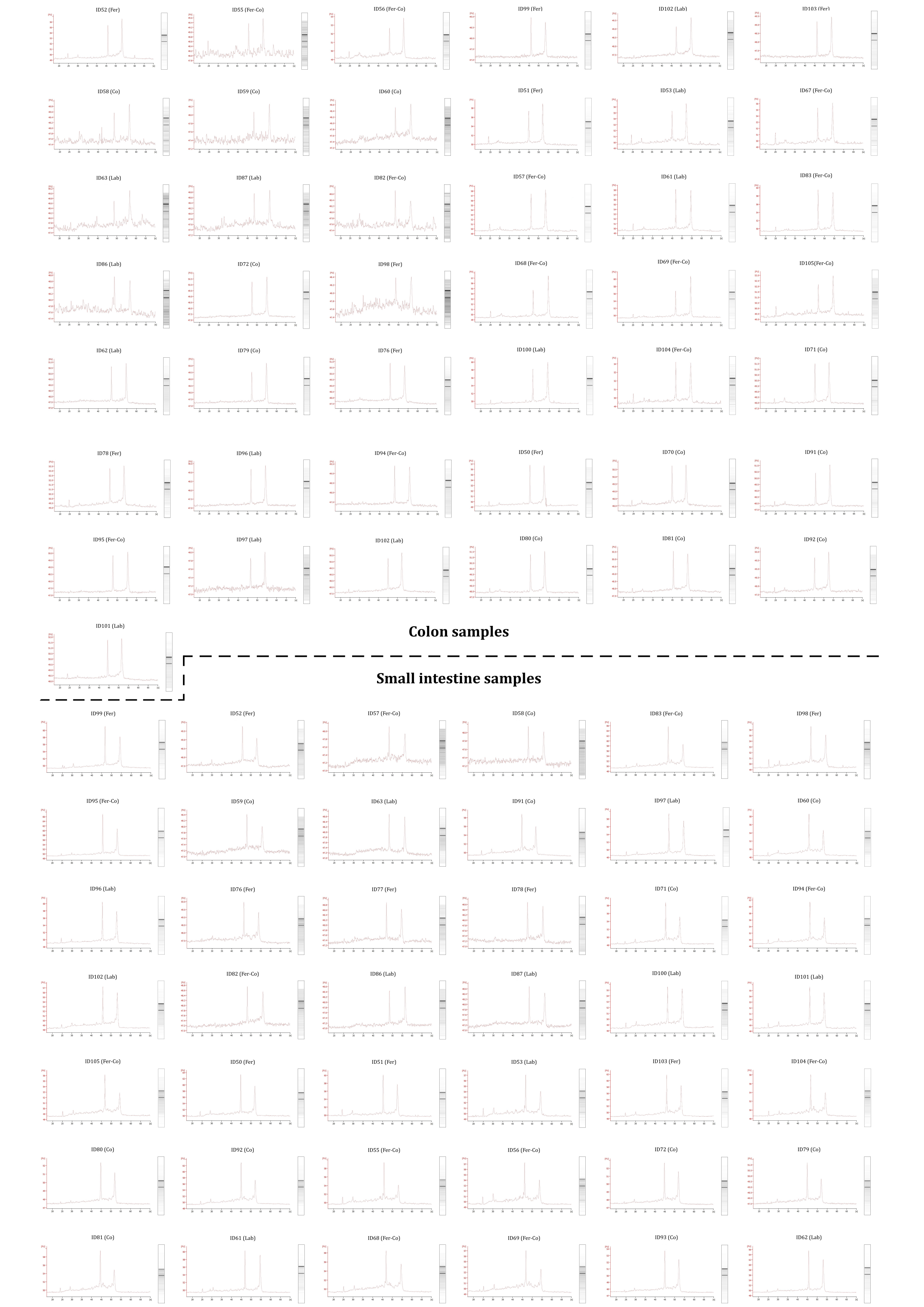

Supplement: kyaf002_suppl_Supplementary_Figures_S1-S6 [file kyaf002_suppl_supplementary_figures_s1-s6.zip › Supplementary Figures S1 to S5/Supplementary Figure S2.png]

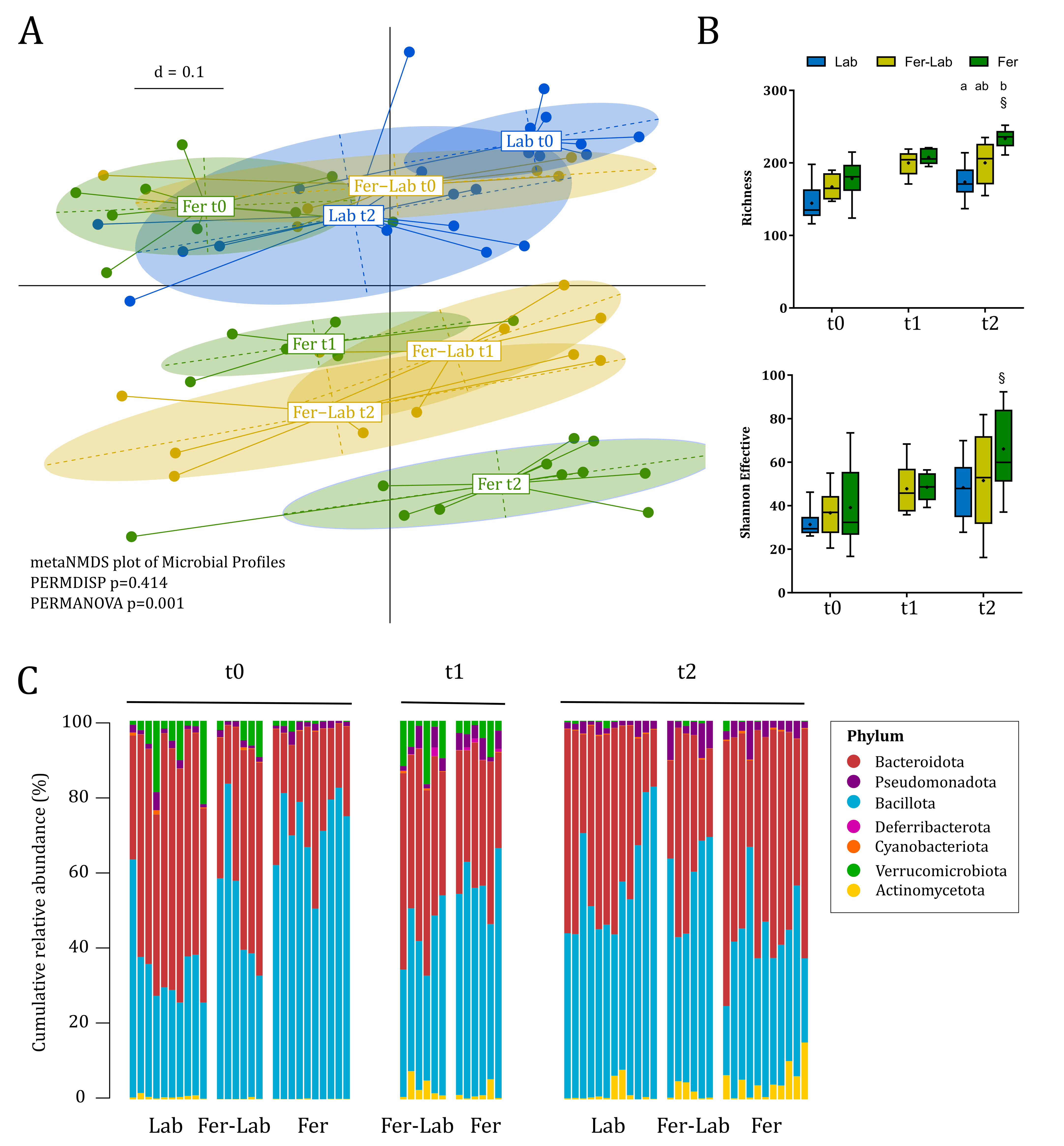

Supplement: kyaf002_suppl_Supplementary_Figures_S1-S6 [file kyaf002_suppl_supplementary_figures_s1-s6.zip › Supplementary Figures S1 to S5/Supplementary Figure S3.png]

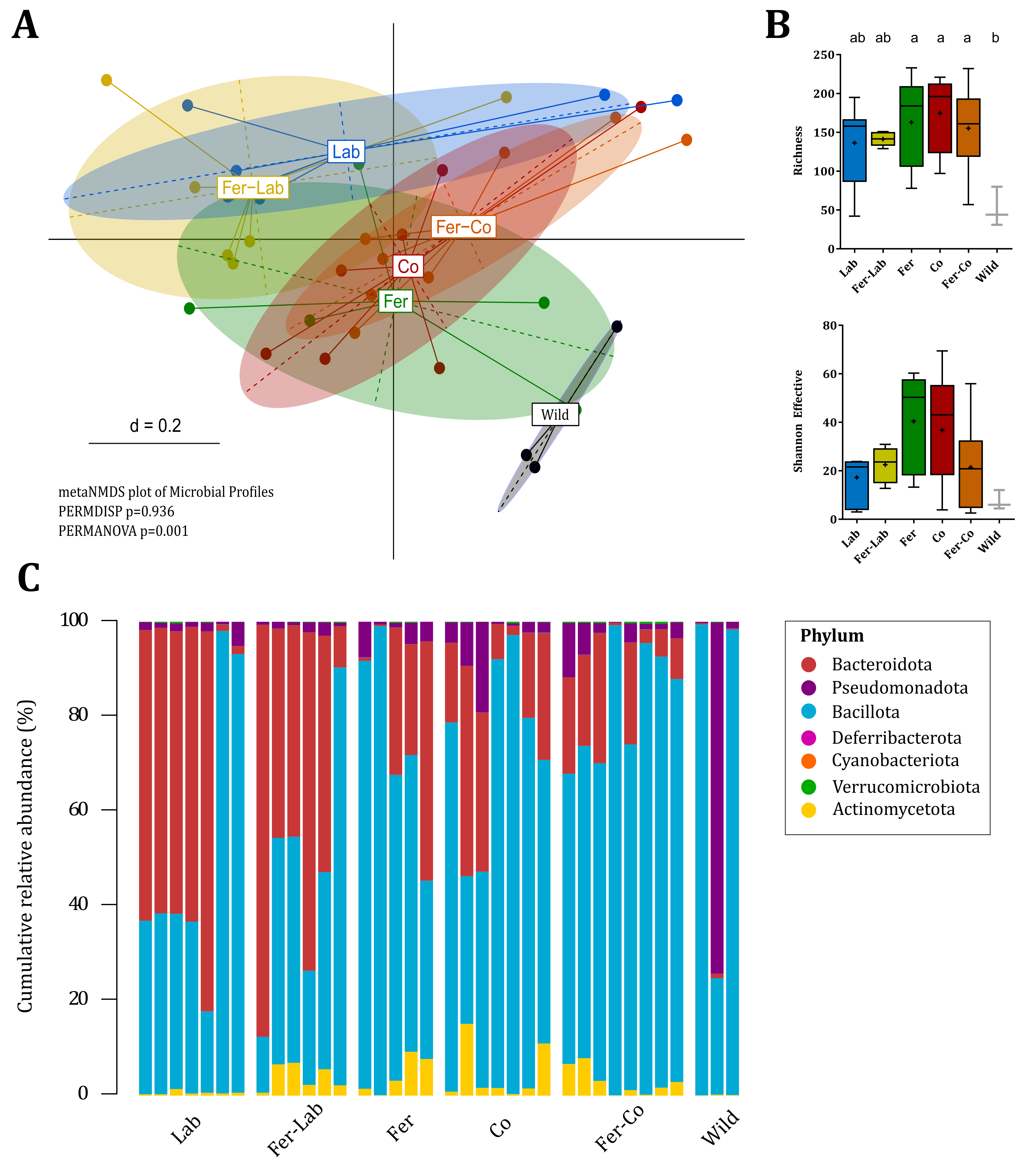

Supplement: kyaf002_suppl_Supplementary_Figures_S1-S6 [file kyaf002_suppl_supplementary_figures_s1-s6.zip › Supplementary Figures S1 to S5/Supplementary Figure S4.png]

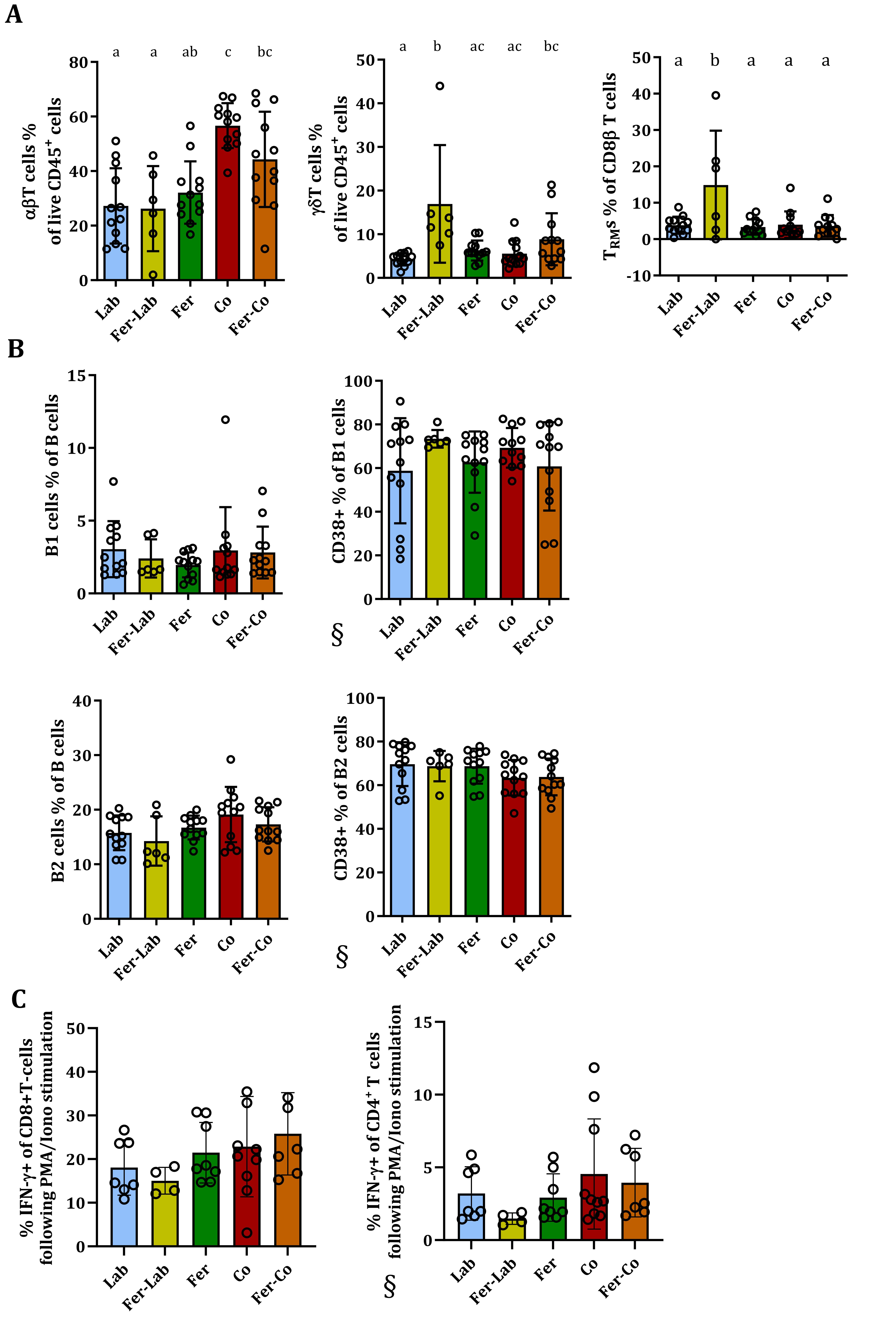

Supplement: kyaf002_suppl_Supplementary_Figures_S1-S6 [file kyaf002_suppl_supplementary_figures_s1-s6.zip › Supplementary Figures S1 to S5/Supplementary Figure S5.png]

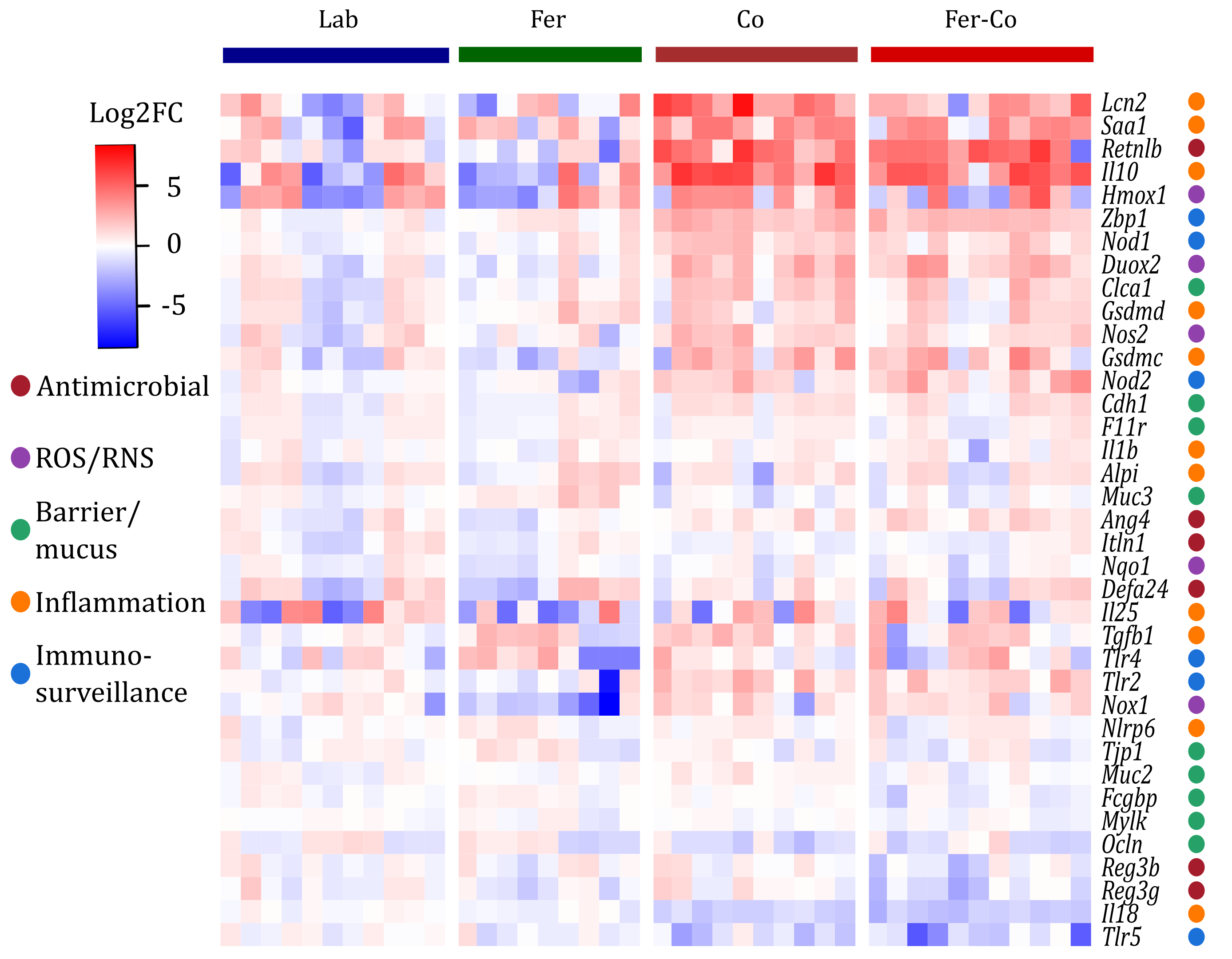

Supplement: kyaf002_suppl_Supplementary_Figures_S1-S6 [file kyaf002_suppl_supplementary_figures_s1-s6.zip › Supplementary Figures S1 to S5/Supplementary Figure S6.png]
